# Electrochemical oxidation of molecular nitrogen to nitric acid – towards a molecular level understanding of the challenges†

**DOI:** 10.1039/d1sc00752a

**Published:** 2021-04-09

**Authors:** Megha Anand, Christina S. Abraham, Jens K. Nørskov

**Affiliations:** Center for Catalysis Theory, Technical University of Denmark Fysikvej Building 311 2800 Kongens Lyngby Denmark jkno@dtu.dk

## Abstract

Nitric acid is manufactured by oxidizing ammonia where the ammonia comes from an energy demanding and non-eco-friendly, Haber–Bosch process. Electrochemical oxidation of N_2_ to nitric acid using renewable electricity could be a promising alternative to bypass the ammonia route. In this work, we discuss the plausible reaction mechanisms of electrochemical N_2_ oxidation (N_2_OR) at the molecular level and its competition with the parasitic oxygen evolution reaction (OER). We suggest the design strategies for N_2_ oxidation electro-catalysts by first comparing the performance of two catalysts – TiO_2_(110) (poor OER catalyst) and IrO_2_(110) (good OER catalyst), towards dinitrogen oxidation and then establish trends/scaling relations to correlate OER and N_2_OR activities. The challenges associated with electrochemical N_2_OR are highlighted.

## Abstract 2

Electrochemical oxidation of N_2_ to HNO_3_ (N_2_OR) is explored in conjunction with parasitic oxygen evolution reaction (OER) on a poor and a good OER catalyst, TiO_2_ and IrO_2_. We develop scaling relations to correlate OER and N_2_OR activities on oxides.

## Graphical abstract

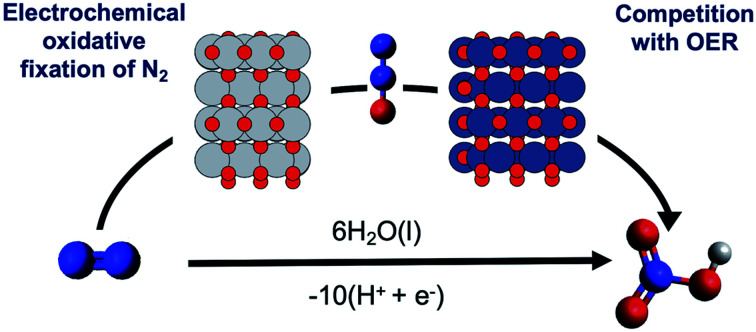

## Introduction

**Fig. 1:**
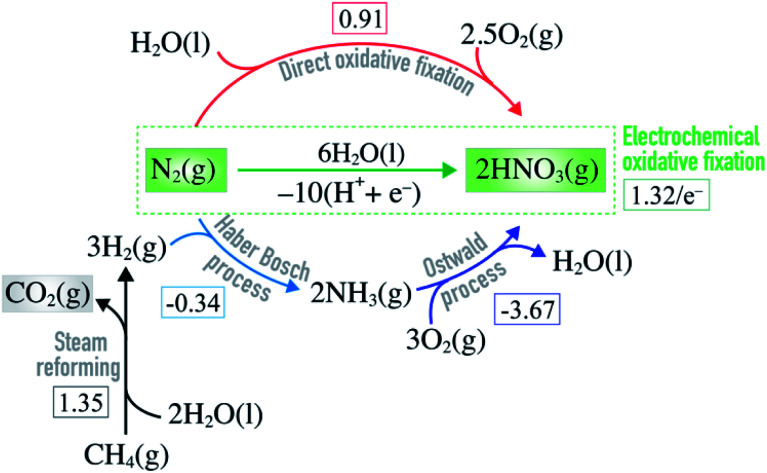
Equations showing how nitric acid is manufactured on industrial scale by combining steam reforming, Haber–Bosch and Ostwald processes. Direct and electrochemical oxidative N_2_ fixation are alternative routes to nitric acid formation. The square brackets contain the free energy of each reaction (in eV) at standard conditions.^8^

Nitric acid is an industrially important compound. It is largely used to make nitrate-based fertilizers that are essential for food production from plants.^1,2^ Without the use of fertilizers it would be impossible to feed the 8 billion human population on earth. Nitric acid used as a basis for nitrate fertilizers is manufactured by oxidizing ammonia using the Ostwald process (Fig. 1), and the ammonia used here comes primarily from the Haber–Bosch (HB) process (N_2_ + 3H_2_ → 2NH_3_).^3,4^ Unfortunately this process requires harsh reaction conditions (*P* ∼ 150 atm and *T* ∼ 700 K) and it is highly energy intensive, using ∼1% of the total global energy consumption. The process has a high carbon-footprint since one of the reactants, H_2_, comes primarily from the steam reforming process where fossil resources react with water to form H_2_ and CO_2_ (Fig. 1). Approximately 1.9 metric tons of CO_2_ is formed per metric ton of NH_3_ produced which contributes significantly to the climate change.^5,6^ Hence, it is highly desirable to bypass the ammonia route and develop a direct and sustainable method for N_2_ fixation such as alternative routes to nitric acid formation.^7^

Direct oxidation of molecular nitrogen provides a moderately endothermic approach to produce nitrogen oxides and, ultimately, nitric acid (Fig. 1).^9,10^ The reaction is however extremely slow at ambient conditions^11^ – a good thing in general, since it helps maintain low concentration of NO_*x*_ and nitric acid in our ecosystem. Only very high temperatures or plasmas enable reasonable reaction rates.^12–19^

Electrochemical oxidative fixation of molecular nitrogen (Fig. 1) appears to be a very attractive approach to drive the endothermic reaction at ambient conditions, where the electricity needed can come from the renewable energy sources making the process sustainable. The reaction has an equilibrium potential of 1.32 eV and previous reports suggest that at pHs above 1.3, the formation of nitrate ions is thermodynamically favoured over the parasitic oxygen evolution reaction for a wide range of potentials.^6^

There is a general lack of natural or artificial electro-catalysts for dinitrogen oxidation.^20^ Recently, a few experimental reports emerged suggesting Pd-decorated MXenes and several oxides as potential electrocatalysts for nitrogen oxidative fixation.^21–24^ There are numerous reports of oxide photo-catalysts for the N_2_ oxidation reaction, prominent among these is TiO_2_, but there is also a great deal of controversy in that field, see ref. 10 for a recent thorough review.

In the present paper, we aim at contributing to the theoretical framework for understanding the electrochemical dinitrogen oxidation reaction (N_2_OR). The goal is to provide design strategies for N_2_OR electro-catalysts both in terms of reaction rates and selectivity towards N_2_ oxidation relative to water oxidation (the oxygen evolution reaction, OER). Building on the work of Medford *et al.*,^25,26^ we first discuss the role of a catalyst in terms of stabilization of key intermediates on the basis of a set of density functional theory (DFT) computations. We contrast two catalysts, a good OER catalyst, IrO_2_, and a poor OER catalyst, TiO_2_. We then identify several possible rate- and selectivity-determining elementary steps and evaluate the corresponding activation energies.

**Fig. 2:**
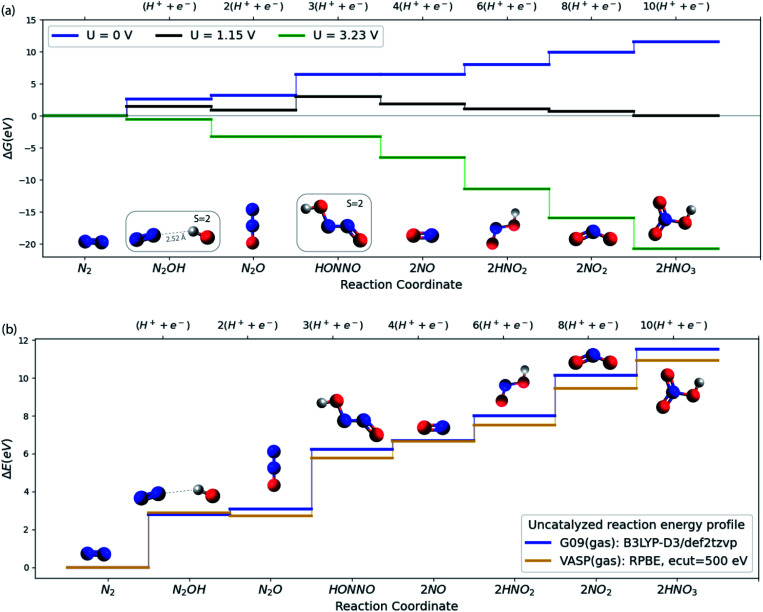
(a) Free energy plot at SMD(H_2_O)/B3LYP-D3/def2tzvp level of theory for N_2_ to HNO_3_ conversion. All the relative free energies (Δ*G*) are evaluated with respect to N_2_(g), H_2_(g) and H_2_O(l). The blue, black and green lines refer to the 0, equilibrium (1.15 V) and the limiting (3.23 V) potentials respectively. (b) Comparison of VASP and G09 energies of the N_2_OR reaction intermediates in vacuum.

As a starting point, consider in Fig. 2a the free energy diagram for a set of intermediates defining the simplest possible pathway for N_2_ oxidation in solution. At the SMD(H_2_O)/B3LYP-D3/def2tzvp level of theory used here (see ESI†/Computational methods section for details), the free energy for this 10-electron electrochemical reaction is 11.5 eV in reasonable agreement with experiment (12.7 eV) when N_2_(g), H_2_(g) and H_2_O(g) are used as the references at standard conditions.^8^ The energetics and the characteristics of the intermediates all agree well with other DFT functionals and experiment as discussed in the ESI.†Fig. 2a illustrates well the difficulty of oxidizing N_2_, and the ease of the opposite reaction, reduction of nitrate to form N_2_.^27,28^ Applying a positive potential can reduce the thermodynamic barriers for N_2_OR, but a very high limiting potential of 3.23 V (*vs.* RHE) is needed in order for all reaction steps to become exergonic.

We now turn to discuss the ways in which a catalyst can facilitate the reaction. We will discuss solid catalysts deposited on an electrode. To this end we need a calculational scheme that can treat semi-infinite solid surfaces. We use VASP^29–32^ with the RPBE exchange–correlation functional,^33^ which is known to provide the best treatment of adsorption properties on solid surfaces^34^ (see ESI†/Computational methods section for details). Fig. 2b compares the gas phase energies of reaction intermediates of the uncatalyzed reaction obtained using the RPBE functional in VASP and the B3LYP-D3 functional in Gaussian 09. For the present purposes, the two functionals provide very similar descriptions of the N_2_OR process. In the following sections, we base our treatment of the electrochemical steps on the RPBE functional. We include entropic terms in the harmonic approximation, (see ESI† for details) in the calculation of the free energies but ignore solvation effects at the surface. We have tested this by studying the electrochemical interface between stoichiometric, defect-free (110) rutile TiO_2_ and explicitly adsorbed water in order to investigate how the presence of water influences the adsorption of the N_2_OR intermediates. We find almost no change in the N_2_OR adsorbate binding energy with the inclusion of explicit water molecules (see ESI†/Solvation section for more details).

**Fig. 3:**
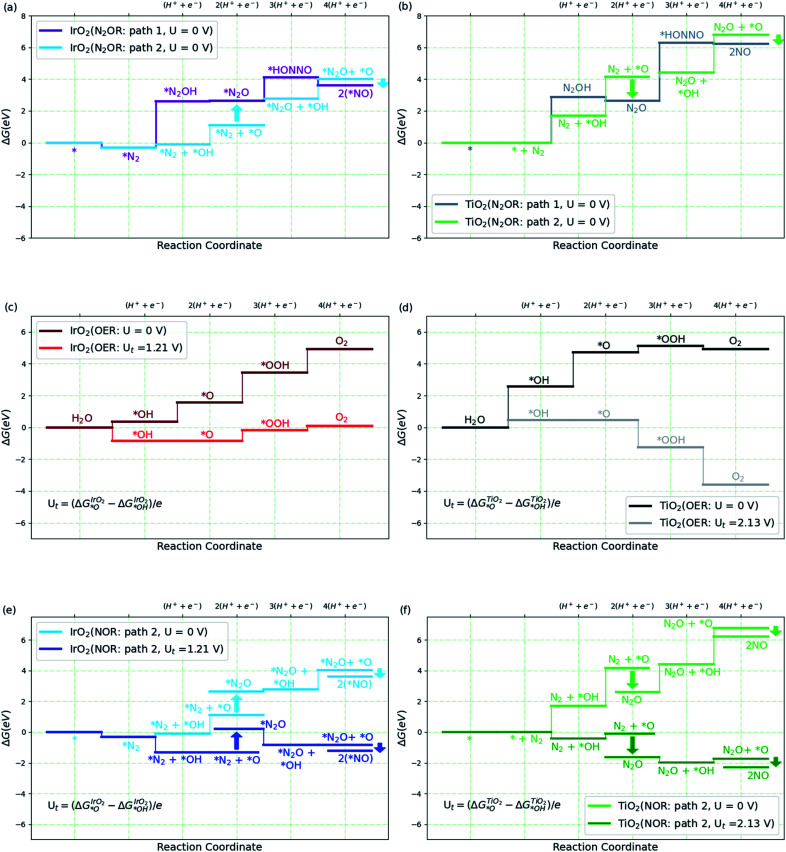
(a and b) Free energy diagram for N_2_ to NO formation through path 1 and path 2 on IrO_2_(110) and TiO_2_(110). (c and d) Free energy diagram for oxygen evolution reaction (OER) on high *O covered IrO_2_(110) and high *OH covered TiO_2_(110) surfaces at two different potentials, *U* and *U*_t_ volts, where *U*_t_ is defined in the plot (see ESI† for coverage details). (e and f) Free energy diagram for N_2_ to NO formation *via* path 2 on IrO_2_(110) and TiO_2_(110) at *U* and *U*_t_ volts. The label 2(*NO) refers to 2 times the NO–IrO_2_(110) system. All the relative free energies (Δ*G*) are evaluated with respect to N_2_(g), H_2_(g) and H_2_O(l).

We then consider the reaction over two transition metal oxide surfaces, IrO_2_ and TiO_2_, both in the rutile structure and in both cases, we consider the (110) facets. Both materials are stable under highly oxidizing conditions, for IrO_2_ at least up to potentials of interest for the oxygen evolution reaction.^9,35–38^ Here we focus on the path to producing NO, since the steps following that are relatively facile even at the equilibrium potential, as shown in Fig. 2a. Fig. 3a and b show the free energy diagram for two pathways for the two surfaces. Path 1 is the one we studied in solution (Fig. 2a), while the new path 2, starts with water oxidation to form adsorbed OH as first discussed by Medford and co-workers.^26^ The formation of adsorbed OH is less endergonic than the first oxidation step of N_2_ to form adsorbed N_2_OH for both surfaces. We therefore concentrate on path 2 in the following sections.

Adsorbed OH (or *OH) can react with N_2_ involving a proton and electron transfer to form N_2_O (*OH + N_2_ → N_2_O + H^+^ + e^−^). Alternatively, *OH can be oxidized further to form adsorbed O, which can react in two ways. It can form O_2_ by direct recombination or react with water in an electrochemical process to form adsorbed OOH and, after another electron and proton transfer, O_2_, Fig. 3c and d. This is the usual oxygen evolution reaction. Alternatively, the adsorbed O can react with N_2_ in a non-electrochemical process *O + N_2_ → *N_2_O, and further, *O + N_2_O → 2*NO (=*N_2_O_2_) (Fig. 3e and f). It can be seen that, as expected, OER is much more facile than N_2_OR for IrO_2_. Indeed, for IrO_2_, *N_2_O is considerably less stable than *O + *N_2_ making the N_2_OR reaction very slow, while OER becomes facile thermochemically at potentials above 1.5 V. On TiO_2_, on the other hand, the adsorbed O is so unstable, that N_2_O formation is highly exergonic. We therefore discuss TiO_2_ in more detail below.

Most of the reaction steps in Fig. 3e and f involve proton transfers from oxygen to water (in acidic conditions). Such barriers have been found to be very small, of the order 0.2 eV, in studies of water oxidation.^39^ We expect the highest barriers to be associated with the activation of N_2_. For the N_2_OR path 2, there are two possible rate determining steps to form N_2_O, the electrochemical pathway, *OH + N_2_ → N_2_O + H^+^ + e^−^ or the purely chemical pathway, *O + N_2_ → N_2_O. In the following, we explore the chemical pathway, including the next chemical step, *O + N_2_O → N_2_O_2_ → 2*NO.

**Fig. 4:**
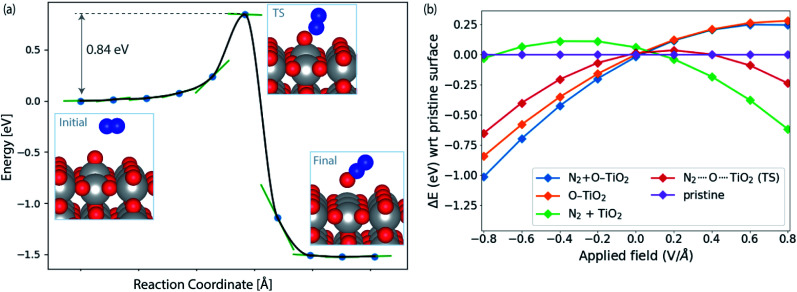
(a) Reaction pathway and barrier for *O + N_2_ → N_2_O formation on TiO_2_(110). (b) Influence of applied field on the energies of initial (N_2_ + O–TiO_2_) and transition state (N_2_⋯O⋯TiO_2_) with respect to the pristine slab (TiO_2_(110)). The orange and green lines correspond to the initial structure (N_2_ + O–TiO_2_) with N_2_ and *O removed from the surface, respectively. See ESI† for details of field computations.

Fig. 4a shows the calculated activation energy for the reaction *O + N_2_ → N_2_O over a TiO_2_(110) surface. A value of 0.84 eV is found at this level of theory. Outside an electrode surface we need to include electric field effects. It can be seen in Fig. 3f that we need to apply a potential of *U*_t_ = (Δ*G*_O_ − Δ*G*_OH_)/e in order for adsorbed O to become thermodynamically stable at the surface. For TiO_2_, this value is *U*_t_ = 2.13 V. If we assume a width of 3 Å for the Helmholtz layer outside the electrode,^40^ this corresponds to a field strength of the order of *E* ∼ *U*_t_/*d* ∼ 0.7 V Å^−1^, depending on the value of the potential of zero charge of the system. In Fig. 4b we show the energy of adsorbed O and the transition state of N_2_O formation as a function of field strength outside a TiO_2_(110) surface. Clearly *O is strongly destabilized while the transition state is stabilized at positive fields. For TiO_2_, the net effect is a reduction of the activation energy of the order 0.5 eV. Finally, in order to evaluate the activation free energy, we need to include the loss of gas phase entropy of N_2_ during the reaction. This would add an energy of the order of 0.6 eV to the free energy barrier for the reaction (see ESI† for more details). The net free energy barrier is thus of the order 1 eV according to this very rough estimate.

An activation free energy of the order 1 eV should give a measurable N_2_OR rate unless the selectivity is low. Based on the model developed in ref. 39 to estimate the activation energy for OER over TiO_2_, the activation energy for oxygen evolution is considerably low. Even given the crudeness of the estimate of the free energy barrier for N_2_OR, this result strongly suggests that N_2_OR through the direct chemical reaction of N_2_ and N_2_O with adsorbed O is difficult over TiO_2_. We cannot rule out that the alternative electrochemical process, *OH + N_2_ → N_2_O + H^+^ + e^−^, will work. That is beyond the present work. We find similar activation barrier of 0.77 eV for the N_2_O_2_ formation (*O + N_2_O → N_2_O_2_).

**Fig. 5:**
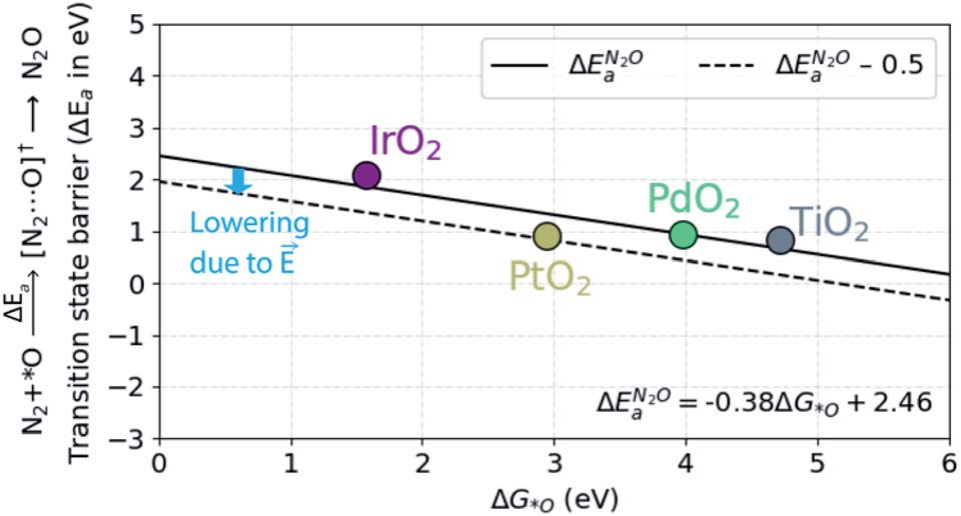
Scaling relationship between Δ*G*_O_ and the barrier (Δ*E*_a_) for the N_2_O formation. The dotted line includes the 0.5 eV lowering of the barrier due to the local electric field that exists in the electric double-layer at the electrode–electrolyte interface in electrochemical reactions.

In order to understand the trends in the chemical N_2_O formation barrier we show in Fig. 5 the variation in the activation energy to form N_2_O with the O adsorption energy including additional oxide surface models. There is a strong linear scaling such that a weaker O adsorption bond gives a lower activation energy. A more facile N_2_OR process would therefore require a catalyst binding O even weaker than TiO_2_. The problem is that such a material would still have low activation energy for OER according to the model in ref. 39.

A more promising strategy towards a high selectivity may be to impede the electrochemical oxygen evolution (OER) step relative to the chemical N_2_OR steps. This can be accomplished by limiting the access to either proton acceptors or electron acceptors (holes). Limited access to proton acceptors can be accomplished by using a non-aqueous solvent with few proton acceptors. A similar strategy has already been used successfully to increase the selectivity of electrochemical N_2_ reduction (where the parasitic reaction is hydrogen evolution).^41–44^ Limited access to holes can for instance be achieved by limiting conductivity to the surface. For TiO_2_ this could be achieved by controlling the thickness of a non-conducting TiO_2_ film on the electrode surface.^45^ Limited access to holes could be what is achieved in photochemical N_2_OR. We note that any lowering of the electrochemical rates will of course lower the overall rate since the first N_2_OR steps are electrochemical.

## Conclusions

In conclusion, we provide a molecular level understanding of the challenges associated with the electrochemical nitrogen oxidation reaction. We analyse the possibility of N_2_OR on an excellent OER catalyst, IrO_2_(110) and a poor OER catalyst TiO_2_(110). Obviously OER supersedes N_2_OR on IrO_2_ and TiO_2_ turns out be a borderline N_2_OR catalyst. We suggest ways to suppress OER in order to promote N_2_OR on oxide surfaces.

## Author contributions

J. K. N. and M. A. conceptualised the paper. M. A. performed all the computations except the aqueous stability computations which were done by C. S. A. All authors contributed towards writing the manuscript.

## Conflicts of interest

There are no conflicts to declare.

## Supplementary Material

SC-012-D1SC00752A-s001

SC-012-D1SC00752A-s002

SC-012-D1SC00752A-s003

SC-012-D1SC00752A-s004

SC-012-D1SC00752A-s005

SC-012-D1SC00752A-s006

SC-012-D1SC00752A-s007

SC-012-D1SC00752A-s008

SC-012-D1SC00752A-s009

SC-012-D1SC00752A-s010

SC-012-D1SC00752A-s011

SC-012-D1SC00752A-s012

SC-012-D1SC00752A-s013

SC-012-D1SC00752A-s014

SC-012-D1SC00752A-s015

SC-012-D1SC00752A-s016

SC-012-D1SC00752A-s017

SC-012-D1SC00752A-s018

SC-012-D1SC00752A-s019

SC-012-D1SC00752A-s020

SC-012-D1SC00752A-s021

SC-012-D1SC00752A-s022

SC-012-D1SC00752A-s023

SC-012-D1SC00752A-s024

SC-012-D1SC00752A-s025

SC-012-D1SC00752A-s026

SC-012-D1SC00752A-s027

SC-012-D1SC00752A-s028

SC-012-D1SC00752A-s029

SC-012-D1SC00752A-s030

SC-012-D1SC00752A-s031

SC-012-D1SC00752A-s032

SC-012-D1SC00752A-s033

SC-012-D1SC00752A-s034

SC-012-D1SC00752A-s035

SC-012-D1SC00752A-s036

SC-012-D1SC00752A-s037

SC-012-D1SC00752A-s038

SC-012-D1SC00752A-s039

SC-012-D1SC00752A-s040

SC-012-D1SC00752A-s041

SC-012-D1SC00752A-s042

SC-012-D1SC00752A-s043

SC-012-D1SC00752A-s044

SC-012-D1SC00752A-s045

SC-012-D1SC00752A-s046

SC-012-D1SC00752A-s047

SC-012-D1SC00752A-s048

SC-012-D1SC00752A-s049

SC-012-D1SC00752A-s050

SC-012-D1SC00752A-s051

SC-012-D1SC00752A-s052

SC-012-D1SC00752A-s053

SC-012-D1SC00752A-s054

SC-012-D1SC00752A-s055

SC-012-D1SC00752A-s056

SC-012-D1SC00752A-s057

SC-012-D1SC00752A-s058

SC-012-D1SC00752A-s059

SC-012-D1SC00752A-s060

SC-012-D1SC00752A-s061

SC-012-D1SC00752A-s062

SC-012-D1SC00752A-s063

SC-012-D1SC00752A-s064

SC-012-D1SC00752A-s065

SC-012-D1SC00752A-s066

SC-012-D1SC00752A-s067

SC-012-D1SC00752A-s068

SC-012-D1SC00752A-s069

SC-012-D1SC00752A-s070

SC-012-D1SC00752A-s071

SC-012-D1SC00752A-s072

SC-012-D1SC00752A-s073

SC-012-D1SC00752A-s074

SC-012-D1SC00752A-s075

SC-012-D1SC00752A-s076

SC-012-D1SC00752A-s077

SC-012-D1SC00752A-s078

SC-012-D1SC00752A-s079

SC-012-D1SC00752A-s080

SC-012-D1SC00752A-s081

SC-012-D1SC00752A-s082

SC-012-D1SC00752A-s083

SC-012-D1SC00752A-s084

SC-012-D1SC00752A-s085

SC-012-D1SC00752A-s086

SC-012-D1SC00752A-s087

SC-012-D1SC00752A-s088

SC-012-D1SC00752A-s089

SC-012-D1SC00752A-s090

SC-012-D1SC00752A-s091

SC-012-D1SC00752A-s092

SC-012-D1SC00752A-s093

SC-012-D1SC00752A-s094

SC-012-D1SC00752A-s095

SC-012-D1SC00752A-s096

SC-012-D1SC00752A-s097

SC-012-D1SC00752A-s098

SC-012-D1SC00752A-s099

SC-012-D1SC00752A-s100

SC-012-D1SC00752A-s101

SC-012-D1SC00752A-s102

SC-012-D1SC00752A-s103

SC-012-D1SC00752A-s104

SC-012-D1SC00752A-s105

SC-012-D1SC00752A-s106

SC-012-D1SC00752A-s107

SC-012-D1SC00752A-s108

SC-012-D1SC00752A-s109

SC-012-D1SC00752A-s110

SC-012-D1SC00752A-s111

SC-012-D1SC00752A-s112

SC-012-D1SC00752A-s113

SC-012-D1SC00752A-s114

SC-012-D1SC00752A-s115

SC-012-D1SC00752A-s116

SC-012-D1SC00752A-s117

SC-012-D1SC00752A-s118

SC-012-D1SC00752A-s119

SC-012-D1SC00752A-s120

SC-012-D1SC00752A-s121

SC-012-D1SC00752A-s122

SC-012-D1SC00752A-s123

SC-012-D1SC00752A-s124

SC-012-D1SC00752A-s125

SC-012-D1SC00752A-s126

SC-012-D1SC00752A-s127

SC-012-D1SC00752A-s128

SC-012-D1SC00752A-s129

SC-012-D1SC00752A-s130

SC-012-D1SC00752A-s131

SC-012-D1SC00752A-s132

SC-012-D1SC00752A-s133

SC-012-D1SC00752A-s134

SC-012-D1SC00752A-s135

SC-012-D1SC00752A-s136

SC-012-D1SC00752A-s137

SC-012-D1SC00752A-s138

SC-012-D1SC00752A-s139

SC-012-D1SC00752A-s140

SC-012-D1SC00752A-s141

SC-012-D1SC00752A-s142

SC-012-D1SC00752A-s143

SC-012-D1SC00752A-s144

SC-012-D1SC00752A-s145

SC-012-D1SC00752A-s146

SC-012-D1SC00752A-s147

SC-012-D1SC00752A-s148

SC-012-D1SC00752A-s149

SC-012-D1SC00752A-s150

SC-012-D1SC00752A-s151

SC-012-D1SC00752A-s152

SC-012-D1SC00752A-s153

SC-012-D1SC00752A-s154

SC-012-D1SC00752A-s155

SC-012-D1SC00752A-s156

SC-012-D1SC00752A-s157

SC-012-D1SC00752A-s158

SC-012-D1SC00752A-s159

SC-012-D1SC00752A-s160

SC-012-D1SC00752A-s161

SC-012-D1SC00752A-s162

SC-012-D1SC00752A-s163

SC-012-D1SC00752A-s164

SC-012-D1SC00752A-s165

SC-012-D1SC00752A-s166

SC-012-D1SC00752A-s167

SC-012-D1SC00752A-s168

SC-012-D1SC00752A-s169

SC-012-D1SC00752A-s170

SC-012-D1SC00752A-s171

SC-012-D1SC00752A-s172

SC-012-D1SC00752A-s173

SC-012-D1SC00752A-s174
