# Supplementary material for: Electrochemical oxidation of molecular nitrogen to nitric acid – towards a molecular level understanding of the challenges
Source: Chem Sci. 2021 Apr 9;12(18):6442–8. doi: 10.1039/d1sc00752a (PMC8115243; doi:10.1039/d1sc00752a)
Supplement: SC-012-D1SC00752A-s001 [file SC-012-D1SC00752A-s001.pdf]

Electronic Supplementary Material (ESI) for Chemical Science.
This journal is © The Royal Society of Chemistry 2021

Supporting Information

Electrochemical Oxidation of Molecular Nitrogen to Nitric Acid - towards a molecular level understanding of the challenges

Megha Anand, Christina S. Abraham, and Jens K. Nørskov*

Center for Catalysis Theory, Technical University of Denmark, Fysikvej Building 311, 2800 Kongens Lyngby, Denmark

E-mail: jkno@dtu.dk

Contents

S1 Uncatalyzed N_2OR Mechanism (Path 1) Discussion **S3**

S2 Computational Methods **S4**

S2.1 Non-periodic DFT computations for molecules S5

S2.2 Periodic DFT computations for surfaces S9

S2.3 Vibrational analysis and ΔG corrections for solids S9

S2.4 Reaction barriers S11

S2.5 Field Effects S12

S3 VASP energies of N_2OR adsorbates on surfaces **S12**

S4 Oxygen Evolution Reaction (OER) **S15**

S4.1 TiO_2(110) S16

S4.2 IrO_2(110) S17

S5 N_2OR reaction intermediates on surfaces **S18**

S6 Reaction barriers and Scaling relationships **S25**

S7 Influence of solvation on N_2OR binding energies **S26**

S8 N_2OR on high coverage TiO_2 surface **S28**

S9 Data for molecules **S35**

S9.1 Gaussian 09 SMD(H_2O)/B3LYP-D3/def2tzvp data S35

S9.2 VASP data S48

References **S54**

S1 Uncatalyzed N_2OR Mechanism (Path 1) Discussion

N_2 is a quite stable and unreactive molecule with the $N\equiv N$ bond energy of 9.8 eV. It is a non-polar closed shell molecule with zero dipole moment. It interacts with H_2O through weak dipole induced dipole interaction to form the first dinitrogen oxidation reaction intermediate N_2OH, where an electron and a proton are also released. N_2OH is an open shell (S=2) vander-waals complex with the hydrogen of the hydroxyl interacting with the one of the nitrogens in the N_2 molecule with $N \cdots H = 2.52$ Å.[1,2] Ideally the oxygen of OH should have been bonded to one of the nitrogens but it is extremely challenging to form such a structure without the use of a catalyst. The $NNOH$ adduct is earlier shown to be 2.17 eV less stable than the reactants $OH + N_2$.[3–5] We find similar thermodynamic barriers (2.64 eV) for the formation of N_2OH from $N_2 + H_2O$.

The next electrochemical step involves removal of a proton and an electron from N_2OH to form stable singlet (S=1) nitrous oxide. Nitrous oxide ($N\equiv N^+{-}O^-$), also known as the laughing gas, is commonly used an oxidizer and has medicinal properties. At the SMD(H_2O)/B3LYP-D3/def2tzvp level of theory, the $N{-}N$ and $N{-}O$ bond distances are 1.119 and 1.183 Å, respectively. These bond distances are in close agreement with the previous experimental and the computational studies.[5–9]

In the next step, a water molecule is added to nitrous oxide form *trans*-hydrogenhyponitric acid $HONNO$ (S=2) where the hydroxyl group is added to the second nitrogen through oxygen.[10] While several conformers of $HONNO$ are possible (Figure S1), the lowest energy structure among all is shown in the figure 2 of the main text.[11] $HONNO$ formation from N_2O has a high thermodynamic barrier of 3.23 eV. This explains why the reverse process to form N_2O from either NOH dimer ($H_2O_2N_2$) or $HON + NO$ is readily observed.[12–14]

$HONNO$ radical readily decomposes into two units of nitrogen monoxide (NO, S=2) and a proton-electron pair. $HONNO$ and dioxohydrazine (N_2O_2) have larger $N \cdots N$ bond and are known to dissociate to monomer nitrogen monoxide with almost no barrier.[15,16] In the next step, NO reacts with water to form nitrous acid (HNO_2).[17,18] HNO_2 oxidizes to form

nitrogen dioxide (NO_2) and a proton-electron pair. The ground state of NO_2 is a doublet with Θ_{ONO}=134.2° and N···O distance is equal to 1.19 Å. The geometry of NO_2 is in close agreement with the previously reported spectroscopic and theoretical structures.[19–25] In the final step, NO_2 hydrolyzes to form nitric acid - HNO_3 where nitrogen has highest possible formal oxidation state of +5. See section S9.1 for Gaussian 09 cartesian coordinates and DFT energies of all the uncatalyzed path 1 dinitrogen oxidation reaction (N_2OR) intermediates.

S2 Computational Methods

Electrochemical reactions like N_2OR in aqueous environment involve protons (or hydroxide anions) and electrons (see Table S1). We use the computational hydrogen electrode (CHE) to estimate the influence of electrode potential on the free energies of reaction intermediates.[26] At standard conditions, the following reaction is in equilibrium:

$$\mathrm{H^+ + e^- \rightleftharpoons \frac{1}{2}\,H_2(g)} \tag{1}$$

$$\therefore \quad \mu(\mathrm{H^+}) + \mu(\mathrm{e^-}) = \frac{1}{2}\,\mu(\mathrm{H_2(g)}) \tag{2}$$

This allows us to obtain the energy of protons and electrons at U=0 V from the DFT energy of gaseous hydrogen. At other potentials U≠0 volts,

$$\mu(\mathrm{H^+}) + \mu(\mathrm{e^-}) = \frac{1}{2}\,\mu(\mathrm{H_2(g)}) - \mathrm{eU} \tag{3}$$

where 'e' is the charge of an electron (equal to 1 in atomic units) and U (versus RHE) is the applied potential in volts at all pHs.

A total of 10 electrons are transferred for completely oxidizing one mole of N_2 to HNO_3.

$$\mathsf{N_2 + 6\,H_2O \xrightarrow{\Delta G_{rxn}} 2\,HNO_3 + 10\,(H^+ + e^-) \qquad \Delta G_{rxn}{=}11.54\ eV{=}1.15\ eV/e^-}$$

Since $\Delta G_{rxn}=-neU_{eqb}$, the equilibrium potential (U_{eqb}) for the reaction is 1.15 Volts. At

Table S1: Reaction mechanism of the electrochemical oxidation of molecular nitrogen to nitric acid (path 1) under acidic pH conditions. 'n' refers to the total number of electrons transferred to N_2 to form an intermediate.

**Acidic conditions (pH<7)**	**n**
$N_2 + H_2O \longrightarrow N_2OH + (H^+ + e^-)$	1
$N_2OH \longrightarrow N_2O + (H^+ + e^-)$	2
$N_2O + H_2O \longrightarrow HONNO + (H^+ + e^-)$	3
$HONNO \longrightarrow 2\,NO + (H^+ + e^-)$	4
$2\,NO + 2\,H_2O \longrightarrow 2\,HNO_2 + (2\,H^+ + e^-)$	6
$2\,HNO_2 \longrightarrow 2\,NO_2 + 2\,(H^+ + e^-)$	8
$2\,NO_2 + 2\,H_2O \longrightarrow 2\,HNO_3 + 2\,(H^+ + e^-)$	10
$N_2 + 6\,H_2O \xrightarrow{\Delta G_{rxn}} 2\,HNO_3 + 10\,(H^+ + e^-)$	10

U_{eqb}=1.15 V, the reactants and products are at equilibrium. The limiting potential (U_{lim}), defined below, is the potential at which all the reaction steps become downhill.

$U_{lim} = \max(\Delta G_1, \Delta G_2, \Delta G_3, \Delta G_4, \Delta G_5/2, \Delta G_6/2, \Delta G_7/2)/e$, where ΔG_n's are the thermodynamic barriers at U=0 volts defined in Table S2. For N_2OR, U_{lim} = 3.23 V corresponds to the energy needed to surmount the highest thermodynamic barrier for the $N_2O \longrightarrow HONNO$ conversion.

Table S2: Thermodynamic barriers for each of the reaction steps in the electrochemical oxidation of N_2 to HNO_3 at potential 0 and U Volts. The charge on the electron, represented as e, is equal to one.

**U = 0 Volts**	**U = U Volts**
$\Delta G_1 = \Delta G(N_2OH)$	$\Delta G_1 = \Delta G(N_2OH) - eU$
$\Delta G_2 = \Delta G(N_2O) - \Delta G(N_2OH)$	$\Delta G_2 = \Delta G(N_2O) - \Delta G(N_2OH) - eU$
$\Delta G_3 = \Delta G(HONNO) - \Delta G(N_2O)$	$\Delta G_3 = \Delta G(HONNO) - \Delta G(N_2O) - eU$
$\Delta G_4 = 2\,\Delta G(NO) - \Delta G(HONNO)$	$\Delta G_4 = 2\,\Delta G(NO) - \Delta G(HONNO) - eU$
$\Delta G_5 = 2\,\Delta G(HNO_2) - 2\,\Delta G(NO)$	$\Delta G_5 = 2\,\Delta G(HNO_2) - 2\,\Delta G(NO) - 2\,eU$
$\Delta G_6 = 2\,\Delta G(NO_2) - 2\,\Delta G(HNO_2)$	$\Delta G_6 = 2\,\Delta G(NO_2) - 2\,\Delta G(HNO_2) - 2\,eU$
$\Delta G_7 = 2\,\Delta G(HNO_3) - 2\,\Delta G(NO_2)$	$\Delta G_7 = 2\,\Delta G(HNO_3) - 2\,\Delta G(NO_2) - 2\,eU$

S2.1 Non-periodic DFT computations for molecules

All molecular species in the main text Figure 2(a) are optimized using the Gaussian 09 suite of programs.[27] We use the B3LYP-D3 functional where D3 refers to the Grimme's D3-dispersion corrections.[28] Alrichs triple-zeta valence polarized basis set, def2tzvp was employed for all atoms.[29] The solvation effects are captured using the Truhlar's SMD implicit solvation model where energies are computed under the influence of the dielectric continuum of H_2O (ϵ=80.0).[30] Vibrational analysis were performed at standard temperature and pressure (see here for theoretical background: http:\\gaussian.com\wp-content\uploads\dl\thermo.pdf) to ensure that the N_2OR intermediates correspond to a minima structure. We considered different conformations of all the intermediates, such as those shown in Figure S1 for N_2OH and HONNO and only report the energetically most favored structures with no imaginary vibrational modes.

Figure S1: Conformers of N_2OH and HONNO. The most favored conformer is enclosed in green boxes.

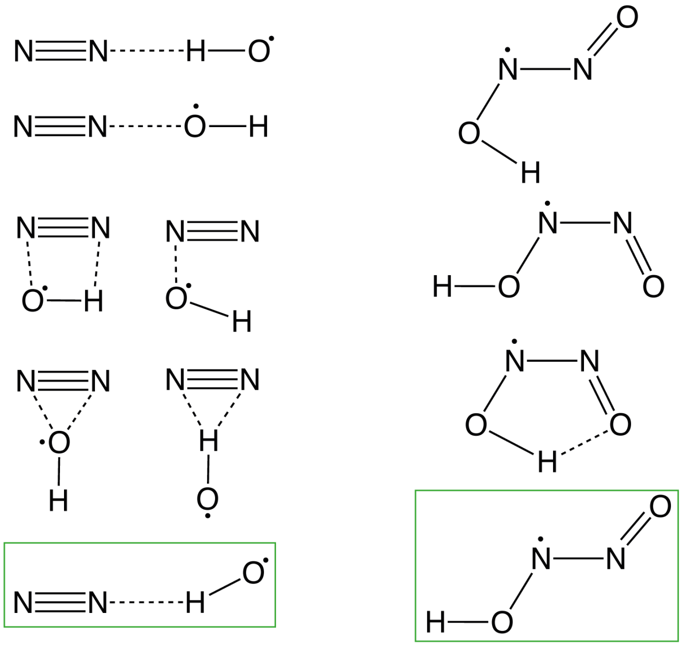

The relative free energies (ΔG) of $N_xO_yH_z(aq)$ (and $N_xO_yH_z(g)$) species are calculated with respect to infinitely separated reactants – $H_2(g)$, $N_2(g)$, and $H_2O(l)$ (see Table S3). We

obtain the free energy of liquid water from the free energy of gaseous water at 0.0313 atm pressure since liquid water is in equilibrium with gaseous water at this pressure and standard temperature. The thermochemistry of $H_2(g)$ and $N_2(g)$ are evaluated at standard temperature and pressure. We show in Table S4 that the free energies obtained using functionals other than B3LYP-D3 are almost similar.

In Gaussian, the implicit solvation computations (SMD(H_2O)/B3LYP-D3/def2tzvp) are done at standard conditions (same as the gas phase computations) with an additional field effect of the dielectric of the solvent. The 1 atm pressure condition is equivalent to 1 mol of an ideal gas in 24.5 litres of volume. Therefore, to convert the free energy of an ideal gas at P=1 atm to condensed phase standard state of 1 M (=1 mol/litre), we needed to apply the correction of RTln(24.5L/1L) (=0.08 eV) to all the energies. This energy is equivalent to the free energy change of compressing an ideal gas from 24.5 L to 1 L volume. We add 0.08 eV correction to the raw DFT free energy of $N_xO_yH_z(aq)$ before evaluating its relative free energies wrt to the $H_2(g)$, $N_2(g)$, and $H_2O(l)$. No such concentration correction was added to the references - $H_2(g)$, $N_2(g)$ and $H_2O(l)$ since they are computed without any implicit solvent effects. The 1 M standard state condition corresponds to a pH=0 and hence all the reported energies of molecular species assume pH=0.

The uncatalyzed (path 1) N_2OR reaction intermediates are also optimized using the periodic DFT code - Vienna Ab initio Simulation Package (VASP)[31–34] which uses plane waves as basis sets. The VASP and G09 energies are compared in Figure 2(b) of the main text. Here the molecules are placed at the center of a large unit cell with a vacuum of 14 Å on all the sides. We use RPBE functional[35] with the plane-wave energy cut-off of 500 eV and Gaussian-level smearing of width 0.05 eV. The molecules are relaxed until the forces are less than 0.05 eV/Å. The PBE projected-augmented wave (PAW) pseudopotentials were used for all the elements in these computations.[36] These settings are similar to what has been used for systems with catalysts (discussed in the next section S2.2). Γ-centered [1,1,1]

*N_2OH did not converge at this level of theory

Table S3: The table below shows how the free energies of each of the reaction intermediates are evaluated at U=0 Volts. G(A) refers to the computed DFT free energies of N_2OR intermediate A(aq) with a correction term for the 1 atm to 1 M standard state conversion (see S2.1 for more details).ΔG(A) (in eV) is the relative free energy of A(aq) at SMD(H_2O)/B3LYP-D3/def2tzvp level of theory wrt H_2(g), N_2(g), and H_2O(l).

$$\begin{aligned}
\Delta G(N_2OH) &= G(N_2OH) - [G(N_2) + G(H_2O) - \tfrac{1}{2} G(H_2)] &&= 2.64 \\
\Delta G(N_2O) &= G(N_2O) - [G(N_2) + G(H_2O) - G(H_2)] &&= 3.19 \\
\Delta G(HONNO) &= G(HONNO) - [G(N_2) + 2\,G(H_2O) - \tfrac{3}{2} G(H_2)] &&= 6.42 \\
\Delta G(NO) &= G(NO) - [\tfrac{1}{2} G(N_2) + G(H_2O) - G(H_2)] &&= 3.20 \\
\Delta G(HNO_2) &= G(HNO_2) - [\tfrac{1}{2} G(N_2) + 2\,G(H_2O) - \tfrac{3}{2} G(H_2)] &&= 3.97 \\
\Delta G(NO_2) &= G(NO_2) - [\tfrac{1}{2} G(N_2) + 2\,G(H_2O) - 2\,G(H_2)] &&= 4.96 \\
\Delta G(HNO_3) &= G(HNO_3) - [\tfrac{1}{2} G(N_2) + 3\,G(H_2O) - \tfrac{5}{2} G(H_2)] &&= 5.77
\end{aligned}$$

Table S4: Relative free energies of each of the intermediates (calculated wrt H_2(g), N_2(g), H_2O(l)) in eV. The def2tzvp basis sets are used with all the functionals. The terms 'gaseous' and 'sol' in brackets refer to the vacuum and condensed phase (SMD(H_2O)) computations, respectively. The grey highlighted numbers are used in the main text.

functional	N_2OH	N_2O	HONNO	NO	HNO_2	NO_2	HNO_3
b3lyp(gaseous)	2.81	3.1	6.46	3.05	4.01	4.75	5.84
b3lyp(sol)	2.57	3.19	6.44	3.2	3.98	4.97	5.8
b3lyp-d3(gaseous)	2.79	3.09	6.44	3.05	4	4.75	5.82
b3lyp-d3(sol)	2.64	3.19	6.42	3.2	3.97	4.96	5.77
b3lyp-d3bj(gaseous)	2.81	3.08	6.41	3.05	3.98	4.74	5.79
b3lyp-d3bj(sol)	2.65	3.18	6.39	3.21	3.96	4.96	5.75
m06(gaseous)	2.9	2.92	6.56	3.21	4.07	4.88	5.83
m06(sol)	2.77	3.02	6.54	3.37	4.04	5.1	5.79
m06-d3(gaseous)	2.9	2.92	6.56	3.21	4.07	4.88	5.83
m06-d3(sol)	2.77	3.02	6.53	3.37	4.04	5.1	5.79
m062x(gaseous)	2.94	3.42	6.94	3.25	4.3	5.29	6.24
m062x(sol)*		3.51	6.9	3.41	4.26	5.5	6.17
m062x-d3(gaseous)	2.94	3.42	6.94	3.25	4.3	5.29	6.24
m062x-d3(sol)	2.76	3.51	6.9	3.41	4.26	5.5	6.17
wb97xd(gaseous)	2.9	3.21	6.62	3.13	4.12	4.93	5.97
wb97xd(sol)	2.71	3.31	6.6	3.29	4.08	5.15	5.91
pbe1pbe(gaseous)	2.87	3.02	6.32	3.12	4.03	4.78	5.76
pbe1pbe(sol)	2.72	3.12	6.30	3.27	3.99	5	5.71

Monkhorst-pack k-point grids were used.[37] The dipole corrections were applied in all three directions for correcting the errors introduced by the periodic boundary conditions.

S2.2 Periodic DFT computations for surfaces

We use VASP[31–34] interfaced with the Atomistic Simulation Environment[38] to perform the periodic DFT computations for modeling the catalytic surfaces. We employed RPBE functional[35] with a plane-wave energy cut-off of 500 eV and Gaussian-level smearing of width 0.05 eV. The (110) catalyst slab of rutile-type single metal oxides MO_2, (M = Ti, Ir) is modeled using a 4-layer 3x2 non-polar and symmetric supercell. The bottom two layers of the slab are fixed at the bulk lattice constant while the rest are allowed to relax until the forces are less than 0.05 eV/Å. Γ-centered [3,2,1] Monkhorst-pack k-point grids were used to sample the first Brioullin zone for these oxides.[37] We chose an optimal size for the slabs, so as to avoid interactions among the large neighboring $N_xO_yH_z$ adsorbates. A minimum vacuum of 15 Å was applied to all the slabs in order to prevent interaction with its periodic images in the z-direction. We have used the PBE projected-augmented wave pseudopotentials for all the elements in these periodic DFT computations.[36] We also apply dipole corrections in the z-direction perpendicular to the slab for all the OER and the low coverage N_2OR DFT computations. We found that high *O and *OH coverages do not influence the binding of N_2OR intermediates (see section S8) and therefore use the low coverage N_2OR data in the manuscript.

S2.3 Vibrational analysis and ΔG corrections for solids

We obtain the free energy corrections to the adsorption/binding energies by performing vibrational frequency analysis on different adsorption modes of N_2OR intermediates on surface. The harmonic oscillator approach as implemented in ASE is used for all the adsorbate-oxide systems where adsorbates are strongly bound to the surface. In this approximation, all the motions of adsorbates - including translations and rotations are treated as harmonic vibra-

tions on surface. The oxide slab is assumed to not contribute to the free energy change upon the adsorption of the N_2OR intermediates. Therefore, only the adsorbate atoms are considered in the numerical evaluation of the Hessian matrix for vibrational frequencies. The 'PV' term is generally small for solids and hence ignored for the adsorbate-oxide systems (see Figure S2).

Figure S2: Figure shows the evaluation of ΔG for one of the bound N_2OR intermediates - $*HONNO$, with respect to the pristine slab and the reference molecules. The terms S, EE, ZPE and 'vib' stand for entropy, electronic, zero-point and vibrational energy, respectively.

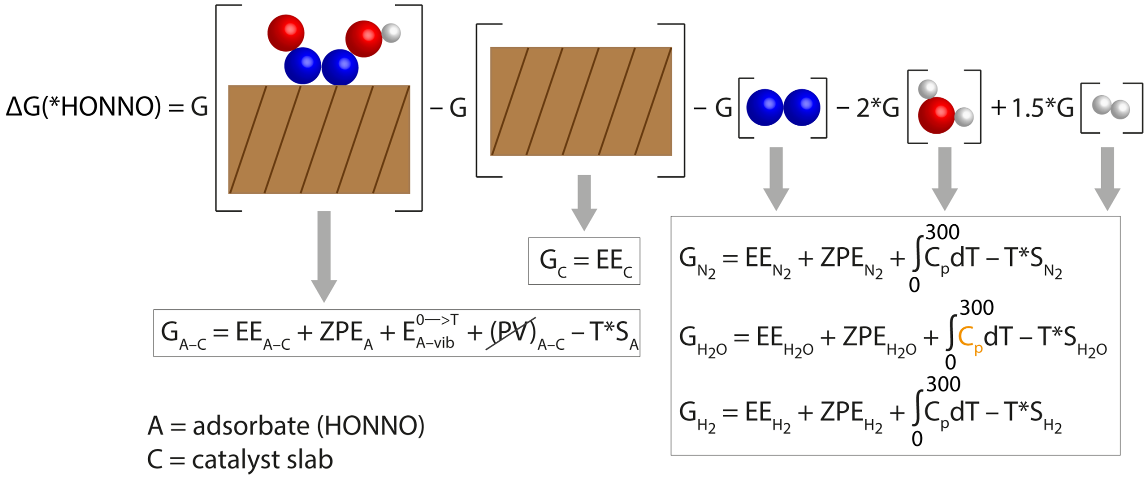

The harmonic oscillator approximation fails for adsorbates that are loosely bound to the surface such as N_2OH, N_2O and NO on $TiO_2(110)$ surface. For these adsorbates we assume negligible interaction with the surface. The adsorbates are treated as free molecules whose ΔG corrections are estimated from the reference molecules (table S5). For example, the ΔG of $TiO_2{-}N_2OH$ is determined as following:

$$G^{corrections}_{TiO_2-N_2OH} = G^{corrections}_{N_2} + G^{corrections}_{H_2O} - 0.5 * G^{corrections}_{H_2} \quad (4)$$

$$\text{where, } G^{corrections}_{N_2/H_2/H_2O} = G_{N_2/H_2/H_2O} - EE_{N_2/H_2/H_2O} \text{ (Figure S2)} \quad (5)$$

$$\therefore \Delta G_{TiO_2-N_2OH} = [E^{DFT}_{TiO_2-N_2OH} + G^{corrections}_{TiO_2-N_2OH}] - [E^{DFT}_{TiO_2} + G_{N_2} + G_{H_2O} - 0.5 * G_{H_2}] \quad (6)$$

The N_2OR reference molecules - $N_2(g)$ and $H_2(g)$ are treated as ideal gases whose thermochemistry is obtained at standard conditions. See this link for the background conceptual de-

tails (https:\\wiki.fysik.dtu.dk\ase\ase\thermochemistry\thermochemistry.html). The entropy of third reference molecule, $H_2O(g)$ is evaluated at standard temperature and 0.0313 atm pressure where it is in equilibrium with $H_2O(l)$. Thus, the ΔG of all the systems involving solids are calculated with respect to $N_2(g)$, $H_2(g)$ and $H_2O(l)$.

We noticed spurious 1-3 small imaginary frequencies below 150 cm^{-1} for some of the N_2OR intermediates on $IrO_2(110)$ and $TiO_2(110)$ surfaces. These frequency modes belonged to soft rotations or translations above the surface that could not be eliminated by either tightening of the convergence criteria or by slightly changing the structure. We replace these imaginary frequencies with a value of 12 cm^{-1} (=0.015 eV) which is a reasonable approximation to estimate the contribution from these shallow vibrational modes.[39] The error arising out of this assumption is considerably small compared to the other factors contributing to the energy and hence unlikely to influence our conclusions.

The free energy corrections for the adsorbed OER reaction intermediates are taken from this paper[40] - 0.35, 0.05 and 0.40 eV for *OH, *O and *OOH respectively. Thus, $\Delta G_{*OH}=\Delta E^{DFT}_{*OH} + 0.35$ and likewise for *O and *OOH. For N_2OR, path 2 intermediates that involve adsorbed OER intermediates, such as $N_2 +$ *OH, we add the corrections as follows:

$$\Delta G_{TiO_2-N_2+*OH} = [E^{DFT}_{TiO_2-N_2+*OH} + G^{corrections}_{N_2} + 0.35] - [E^{DFT}_{TiO_2} + G_{N_2} + G_{H_2O} - 0.5 * G_{H_2}] \tag{7}$$

where we added $*$ in front of OH in the subscript of ΔG to emphasize that the OH is bound to the TiO_2 surface while N_2 is not.

S2.4 Reaction barriers

We locate the pathway and barrier for $N_2 + *O \longrightarrow N_2O$ conversion on different surfaces using the nudged elastic band (NEB) computations. We first interpolate 4-9 intermediate images between the initial and final states and the perform NEB computations using the climbing-image method.[41] The structures are relaxed until the forces converge below 0.05

eV/Å. The FIRE (Fast Inertial Relaxation Engine) force optimizer was most effective in converging the reaction pathways. We obtain the barriers on four surfaces - $TiO_2(110)$, $IrO_2(110)$, $PtO_2(110)$ and $PdO_2(110)$. The computational VASP-tags for $PdO_2(110)$ are same as those of $TiO_2(110)$ and $IrO_2(110)$, discussed earlier in section S2.2. For $PtO_2(110)$, all VASP tags are kept same except the kpoints. We use Γ-centered [3,3,1] Monkhorst-pack k-point grids to sample the first Brioullin zone of $PtO_2(110)$. The same NEB method was used to get the $N_2O + {*O} \longrightarrow N_2O_2 \longrightarrow 2\,NO$ formation on TiO_2.

S2.5 Field Effects

In Figure 4b of the main text, we assess the influence of electric field on the initial and the transition state for the $N_2 + {*O}$ to N_2O conversion on $TiO_2(110)$. We use Quantum Espresso[42] to perform single point energy (SPE) computations on the converged initial state (IS) and transition state (TS) geometries from VASP. The SPE computations are spin-polarized done using RPBE functionals. The plane wave energy and density cutoff of 500 and 5000 eV, respectively, are used. The atoms are represented using the Standard solid-state pseudopotentials (SSSP) and Γ-centered [3,2,1] Monkhorst-pack k-point grids are used to sample the Brioullin zone. The y-axis in Figure 4b plots how the energy (ΔE) of the $adsorbate{-}TiO_2$ system changes in comparison with TiO_2 under the influence of field. The ΔE for an $adsorbate{-}TiO_2$ system at field, x V/Å is obtained as follows:

$$\begin{aligned} \Delta E_{ads-TiO_2}(x\ V/\text{Å}) =& [E_{ads-TiO_2}(x\ V/\text{Å}) - E_{ads-TiO_2}(0\ V/\text{Å})] \\ & -[E_{TiO_2}(x\ V/\text{Å}) - E_{TiO_2}(0\ V/\text{Å})] \end{aligned} \quad (8)$$

S3 VASP energies of N_2OR adsorbates on surfaces

We provide all the remaining VASP energies in the section S9.2.

Table S5: Energy components and free energies (in eV) of reference molecules obtained using DFT computations at standard temperature. We use 1 atm pressure for H_2 and N_2 while 0.0313 atm for H_2O.

Molecule	G	ZPE	T*S	$\int_0^{300K} C_p dT$
H_2	-7.092	0.271	0.407	0.09
H_2O	-14.695	0.567	0.675	0.104
N_2	-17.556	0.149	0.597	0.091

Table S6: All energies reported in the main text and the SI for the $IrO_2(110)$ system. The $E_{A-vib}^{0\rightarrow 300K}$ term is same as $\int_0^{300} C_{v,\,A-harmonic\,vib}(T)dT$ where A refers to the adsorbate molecule. The highlighted free energies, lowest of all binding modes, are used in Figure 3 plots in the main text.

species	E_{DFT}	G	zpe	TS	$E_{A-vib}^{0\rightarrow 300K}$	ΔG
IrO_2	-986.346636	-986.347	0	0	0	0
IrO_2-N_2	-1004.377324	-1004.220	0.227	0.137	0.067	-0.32
IrO_2-N_2OH-a	-1012.622705	-1012.149	0.597	0.234	0.111	2.90
IrO_2-N_2OH-b	-1012.981841	-1012.447	0.62	0.178	0.093	2.61
IrO_2-N_2+OH	-1015.632337	-1015.151	0.587	0.224	0.118	-0.10
IrO_2-N_2O-a	-1006.691071	-1006.495	0.280	0.176	0.092	5.01
IrO_2-N_2O-b	-1008.68898	-1008.573	0.318	0.315	0.113	2.93
IrO_2-N_2O-c	-1009.129606	-1008.869	0.342	0.178	0.097	2.64
IrO_2-N_2+O	-1010.616708	-1010.403	0.302	0.186	0.098	1.10
$IrO_2-HONNO-a$	-1018.440526	-1017.840	0.718	0.241	0.123	4.81
$IrO_2-HONNO-b$	-1018.77155	-1018.126	0.739	0.21	0.117	4.53
$IrO_2-HONNO-c$	-1018.826259	-1018.199	0.727	0.216	0.116	4.46
$IrO_2-HONNO-d$	-1018.897154	-1018.268	0.724	0.209	0.114	4.39
$IrO_2-HONNO-e$	-1019.183508	-1018.548	0.722	0.202	0.115	4.11
IrO_2-N_2O+OH	-1020.427996	-1019.872	0.699	0.298	0.155	2.78
IrO_2-NO-a	-999.5637201	-999.470	0.168	0.151	0.077	3.26
IrO_2-NO-b	-1001.005409	-1000.852	0.214	0.124	0.063	1.88
IrO_2-NO-c	-1001.047574	-1000.920	0.189	0.128	0.067	1.81
IrO_2-N_2O+O	-1015.409632	-1015.092	0.421	0.23	0.127	4.02

Table S7: All energies reported in the main text and the SI for the $IrO_2(110)$ system. The $E^{0\to300K}_{A-vib}$ term is same as $\int_0^{300} C_{v,\,A-\mathrm{harmonic\,vib}}(T)dT$ where A refers to the adsorbate molecule. The highlighted free energies, lowest of all binding modes, are used in Figure 3 plots in the main text. The correction terms for loosely bound adsorbates are obtained from those of free molecules as discussed in section S2.3.

species	E_{DFT}	G	zpe	TS	$E^{0\to300K}_{A-vib}$	ΔG
TiO_2	-1251.680983	-1251.681	0	0	0	0
TiO_2-N_2OH-a	-1277.176334	-1277.514				2.87
TiO_2-N_2+OH	-1278.665371	-1278.672				1.71
TiO_2-N_2O-a	-1273.903719	-1274.219				2.62
TiO_2-N_2O-b	-1273.930206	-1274.245				2.59
TiO_2-N_2O-c	-1273.907502	-1274.223				2.62
TiO_2-N_2+O	-1272.379787	-1272.687				4.15
$TiO_2-HONNO-a$	-1281.937897	-1281.509	0.659	0.399	0.169	6.48
$TiO_2-HONNO-b$	-1281.821094	-1281.287	0.676	0.276	0.134	6.70
$TiO_2-HONNO-c$	-1282.05964	-1281.583	0.675	0.364	0.166	6.40
$TiO_2-HONNO-d$	-1282.048886	-1281.598	0.672	0.389	0.168	6.39
$TiO_2-HONNO-e$	-1282.143178	-1281.690	0.671	0.38	0.162	6.30
TiO_2-N_2O+OH	-1283.600726	-1283.566				4.42
TiO_2-NO-a	-1264.738022	-1264.875				3.19
TiO_2-NO-b	-1264.738878	-1264.875				3.19
TiO_2-NO-c	-1264.812442	-1264.949				3.11
TiO_2-N_2O+O	-1277.392577	-1277.658				6.78

S4 Oxygen Evolution Reaction (OER)

There are excellent reviews and research articles discussing OER using a variety oxides including TiO_2 and IrO_2.[43–46] Among all these oxides, Iridium oxide is proven to be one of the best OER electrocatalysts.[47–51] It has been shown that the *OH, *O and *OOH adsorbate binding energies on the oxides can be correlated with the observed experimental OER trends/activities.[52–54] In this section, we show the OER adsorbate binding energies on different oxides.

S4.1 $TiO_2(110)$

Figure S3: OER reaction intermediates on $TiO_2(110)$ at different coverages. The numbers are the differential ΔG of each of the adsorbates in eV.

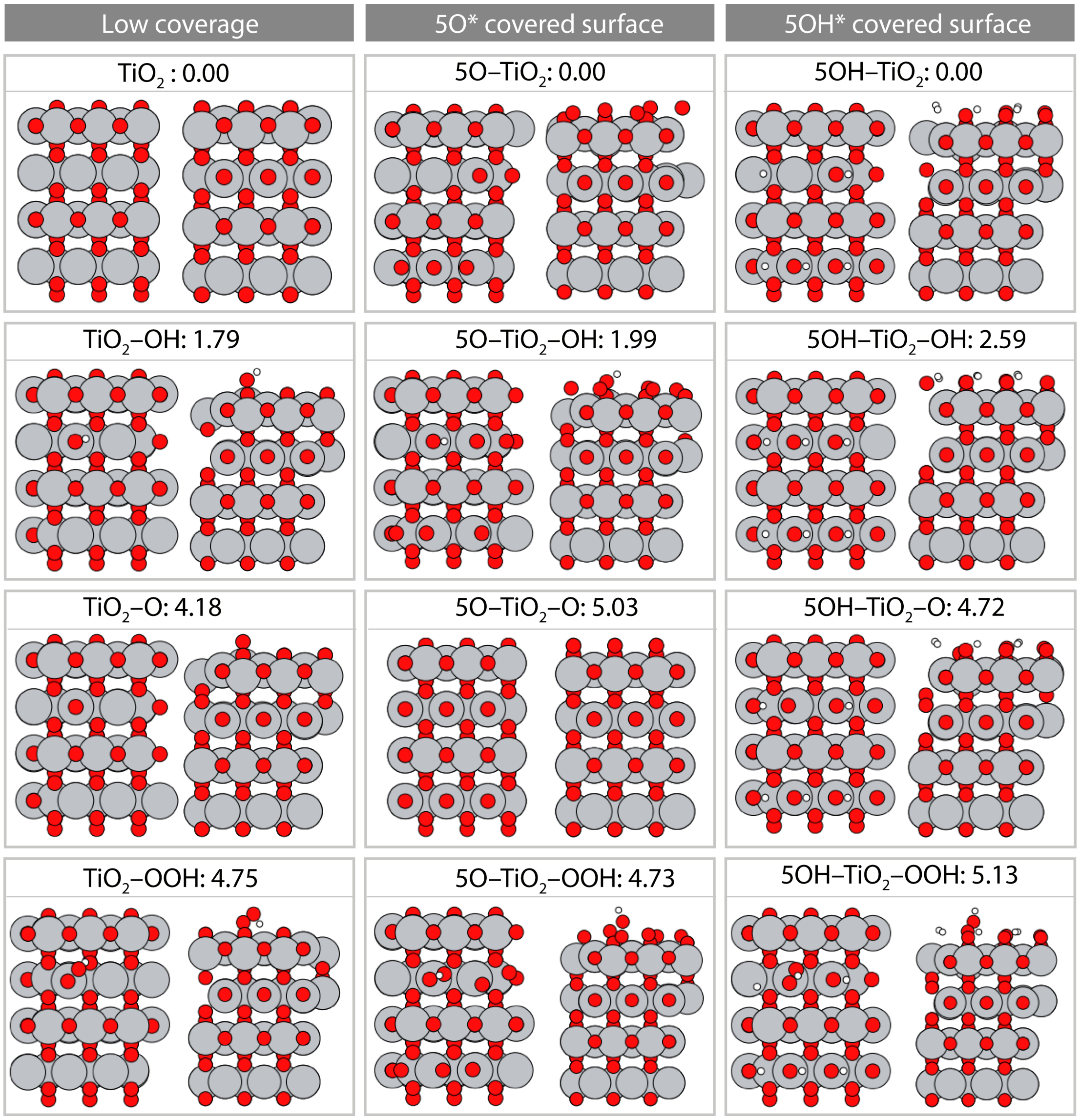

Table S8: Energies (in eV) of OER reaction intermediates on $TiO_2(110)$. At higher potentials, 5 OH* covered surface is energetically most favored. The highlighted numbers are use din the main text figure.

	ΔG_{OH}	ΔG_O	ΔG_{OOH}	$\Delta G_O - \Delta G_{OH}$	$\Delta G_{OOH} - \Delta G_{OH}$
Low coverage	1.79	4.18	4.75	2.39	2.96
5 O* coverage	1.99	5.03	4.73	3.04	2.74
5 OH* coverage	2.59	4.72	5.13	2.13	2.54
published[55,56]	2.28	4.87	5.26	2.59	2.98

S4.2 $IrO_2(110)$

Figure S4: OER reaction intermediates on $IrO_2(110)$ surface at different coverages. The numbers are differential ΔG of each of the adsorbates in eV.

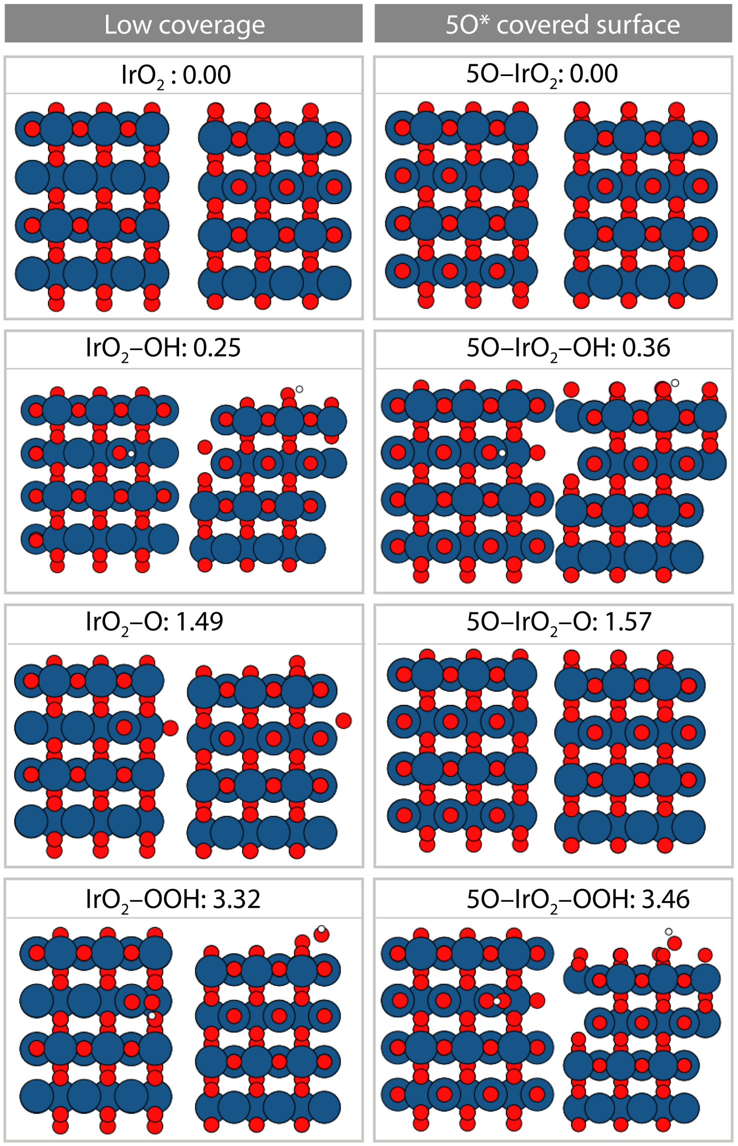

Table S9: Energies (in eV) of OER reaction intermediates on $IrO_2(110)$. The highlighted numbers are used in the main text figure.

	ΔG_{OH}	ΔG_{O}	ΔG_{OOH}	$\Delta G_{O} - \Delta G_{OH}$	$\Delta G_{OOH} - \Delta G_{OH}$
Low coverage	0.25	1.49	3.32	1.24	3.07
5 O* coverage	0.36	1.57	3.46	1.21	3.10
published[52,56]	0.25	1.68	3.42	1.43	3.17

We use the 5 OH* and 5 O* covered energies of $TiO_2(110)$ and $IrO_2(10)$, respectively in Figure 3(c-d) of the main text. Table S10 lists the OER adsorbate energies on $PdO_2(110)$ and $PtO_2(110)$.

Table S10: Energies of OER reaction intermediates on other oxides shown in figure 5 in the main text.

	ΔG_{OH}	ΔG_{O}	ΔG_{OOH}	$\Delta G_{O} - \Delta G_{OH}$	$\Delta G_{OOH} - \Delta G_{OH}$
PdO_2	1.96	3.98	4.77	2.02	2.81
PtO_2	1.11	2.94	4.18	1.83	3.07

S5 N_2OR reaction intermediates on surfaces

The N_2OR intermediates can bind to the catalyst surface in various ways adapting different conformations. We considered each of these forms at the ontop and bridging metal-CUS sites of the $TiO_2(110)$ and $IrO_2(110)$ surfaces. However, not all binding modes/conformations are stable on a given surface and hence do not converge to the structure as desired. In the section below we first show all the possible binding modes for adsorbates - $*N_2OH$, $*N_2O$, *HONNO and *NO that were considered (Figure S5 - S8), followed by the unique structures from the DFT computations that converged (Figure S9 - S12).

N_2OH is extremely difficult to form without any catalyst. N_2OH does not bind to TiO_2 while IrO_2 stabilizes N_2OH in two forms shown here(a-b). On both TiO_2 and IrO_2 it is energetically favorable for N_2OH to dissociate as N_2 and OH.

Similar to N_2OH we consider various different binding modes for N_2O as well (Figure S6). We show the unique converged structures in Figure S10. N_2O is a stable linear molecule and

Figure S5: Possible binding modes and conformations of N_2OH on a catalyst surface.

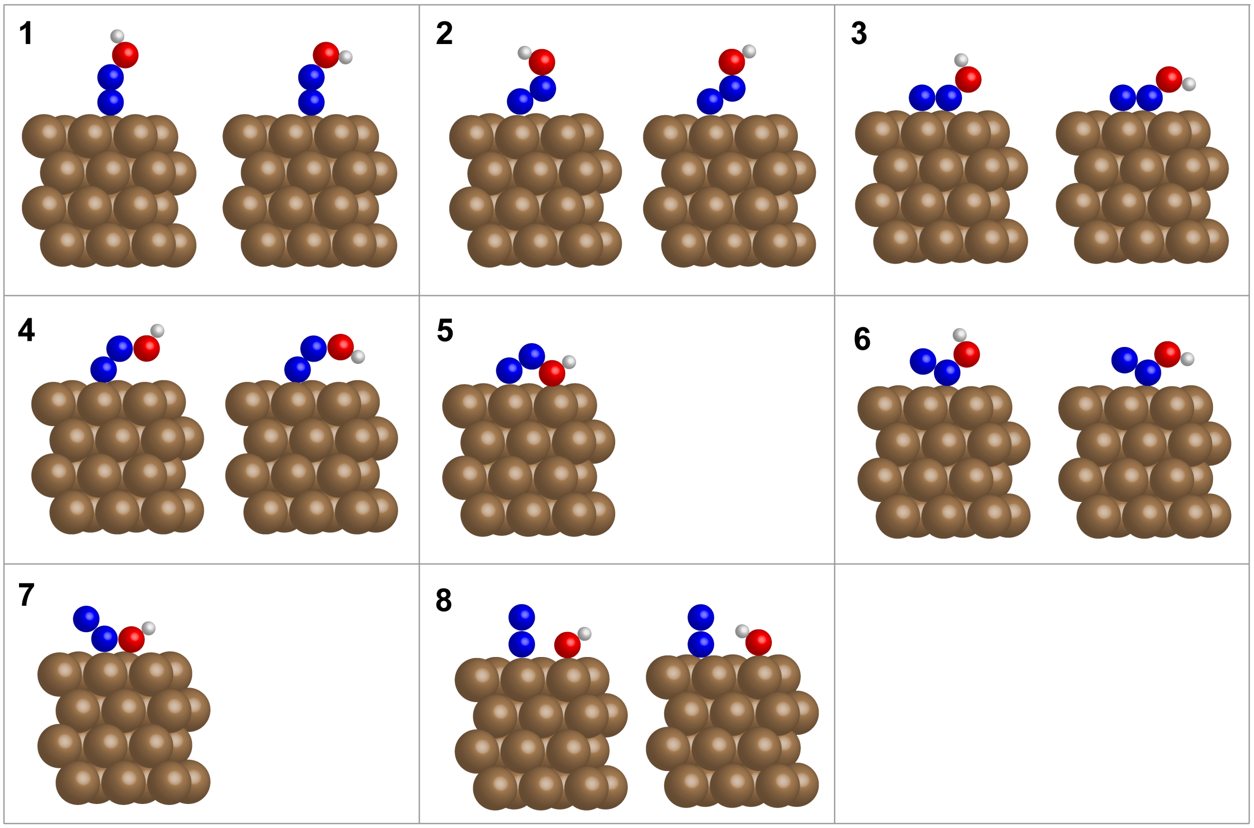

Figure S6: Possible binding modes and conformations of N_2O on a catalyst surface.

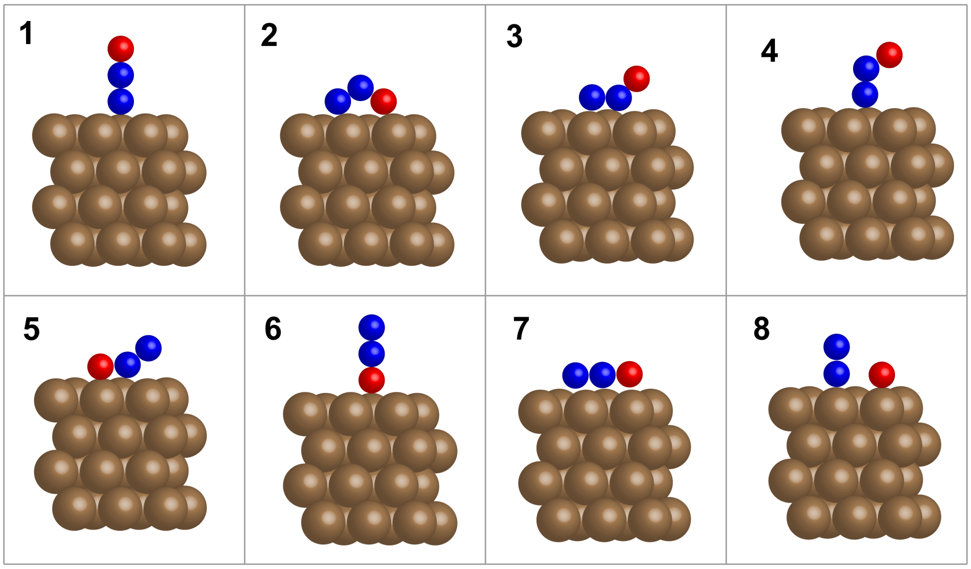

Figure S7: Possible binding modes and conformations of HONNO on a catalyst surface.

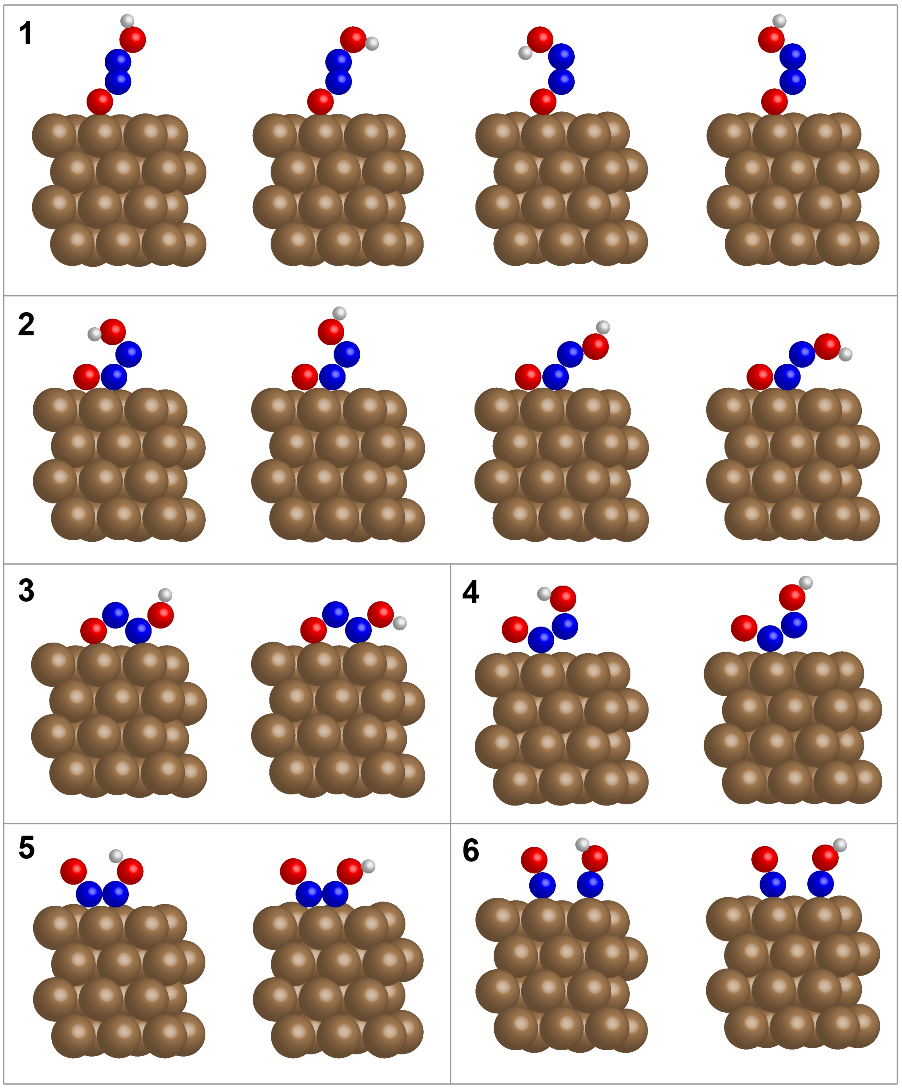

Figure S8: Possible binding modes and conformations of NO on a catalyst surface.

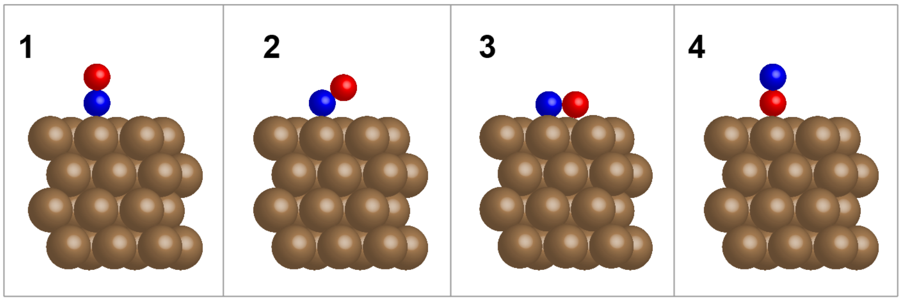

Figure S9: Unique binding modes and conformations of N_2OH on $TiO_2(110)$ and $IrO_2(110)$ surfaces obtained from the DFT computations.

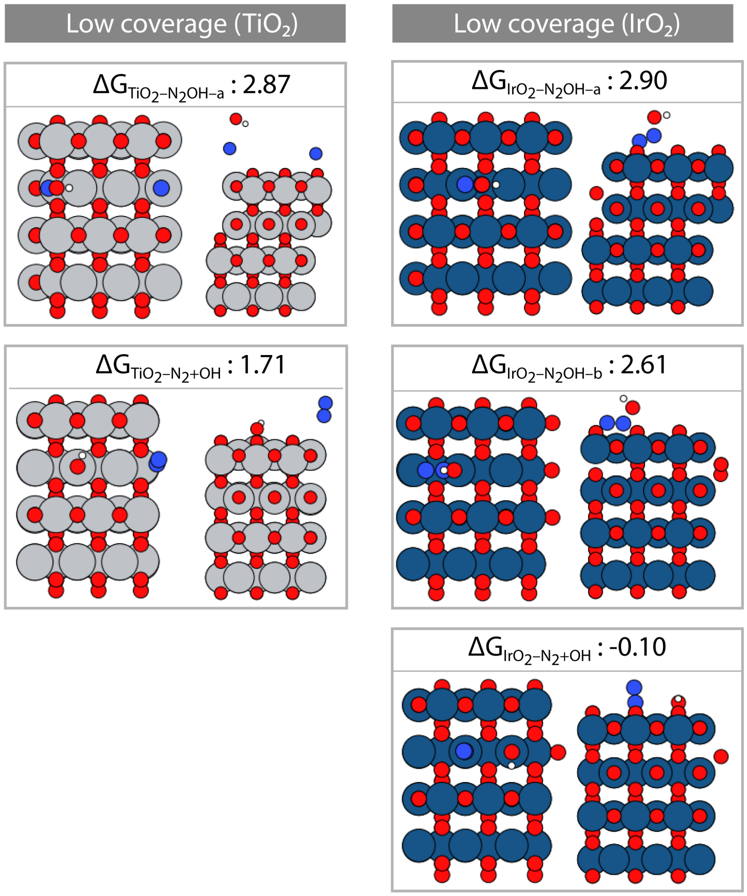

TiO_2 surface is not reactive enough to deform it. On IrO_2, though N_2O bends, it is much higher in energy (Figure S10a).

Figure S10: Unique binding modes and conformations of N_2O on $IrO_2(110)$ and $TiO_2(110)$ surfaces obtained using DFT computations.

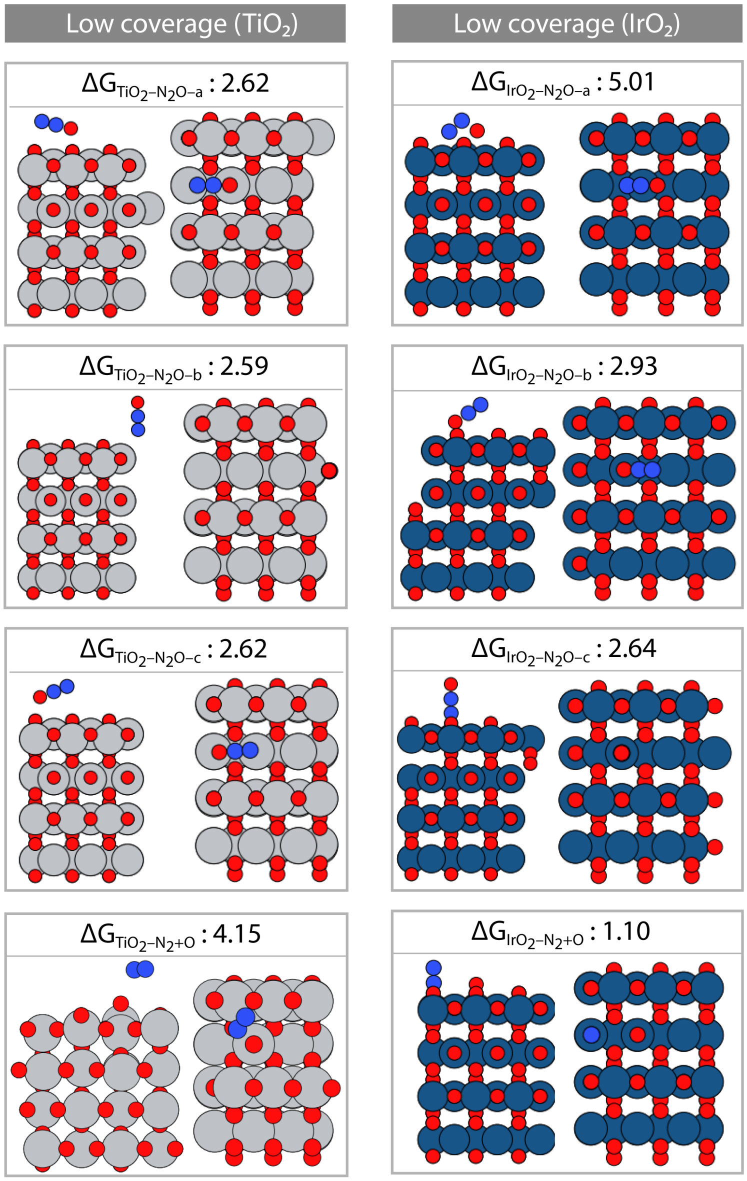

Figure S11: Different binding modes of HONNO adsorbates on $IrO_2(110)$ and $TiO_2(110)$ surfaces obtained using DFT computations. We also show the N_2O + OH adsorbate on $TiO_2(110)$ and $IrO_2(110)$ for the N_2OR path 2 mechanism discussed in the main text.

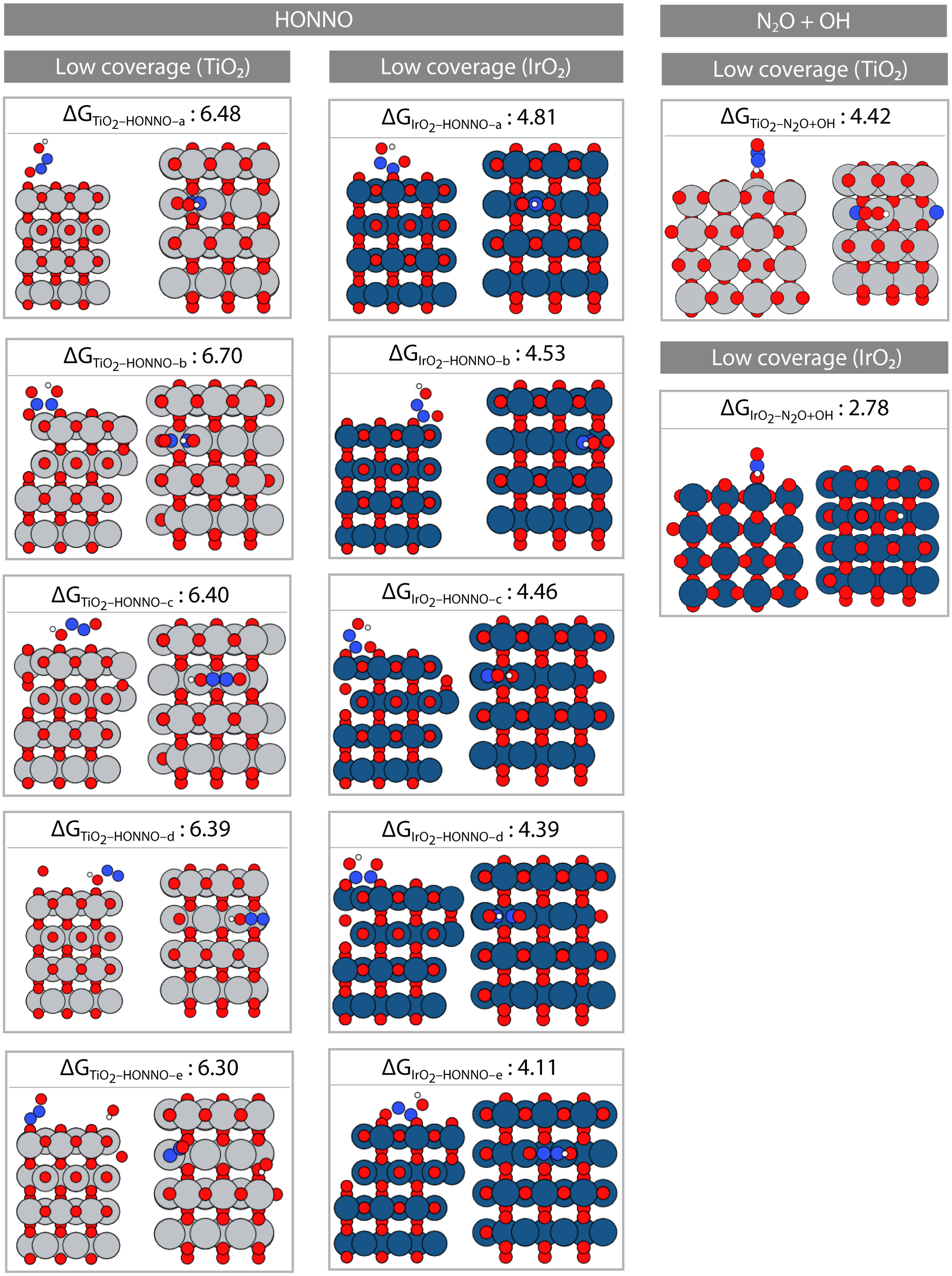

Figure S12: Unique binding modes and conformations of NO on $IrO_2(110)$ and $TiO_2(110)$ surfaces obtained using DFT computations.

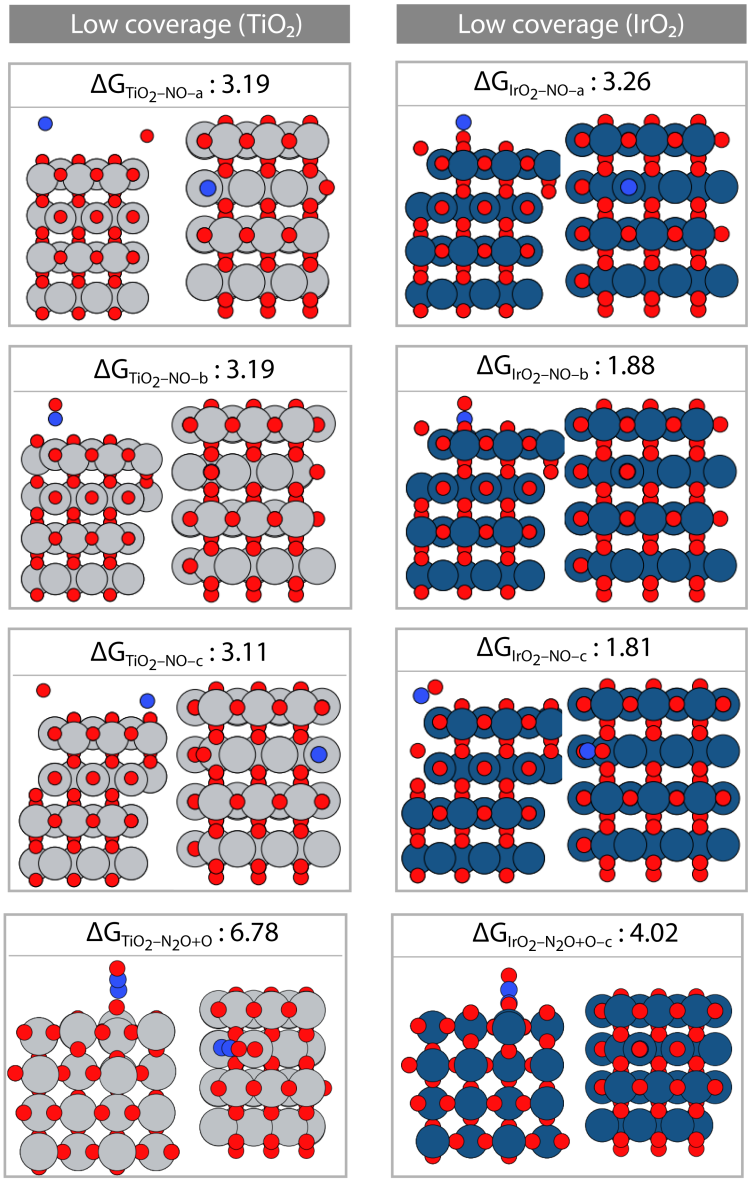

S6 Reaction barriers and Scaling relationships

Table S11: Energy barriers (in eV) for the $(*)N_2 + *O \longrightarrow N_2O$ step on different surfaces. $\Delta E_a = E_{TS} - E_{IS}$ where TS and IS refers to the initial and the transition state in the reaction, $(*)N_2 + *O \longrightarrow N_2O$. The *O binding free energies on the same surface is included in the second column.

Catalyst	ΔE_a	ΔG_O
TiO_2(110)	0.84	4.72
PtO_2(110)	0.91	2.94
IrO_2(110)	2.10	1.57
PdO_2(110)	0.95	3.98

We further verify that all the transition states in this table have only one imaginary frequency. If we further increase the *OH or *O coverage on TiO_2, the adsorbed O combines with the surface or interstitial O to form O_2 making N_2OR difficult on the TiO_2 surface. For surfaces where N_2 is not adsorbed, there is a loss of energy of the order of 0.6 eV due to the loss of entropy of N_2 in the transition state. We use simple linear regression to obtain the scaling relationship between ΔE_a and ΔG_O. The images of the initial, transition and final states of all other transition states discussed in the manuscript are shown in the next figure (Figure S13).

Figure S13: NEB results for chemical N_2O formation on different surfaces. The IS, TS and FS refer to the initial, transition and final state respectively. The last figure shows the NEB for $N_2O + *O \longrightarrow N_2O_2$ on $TiO_2(110)$.

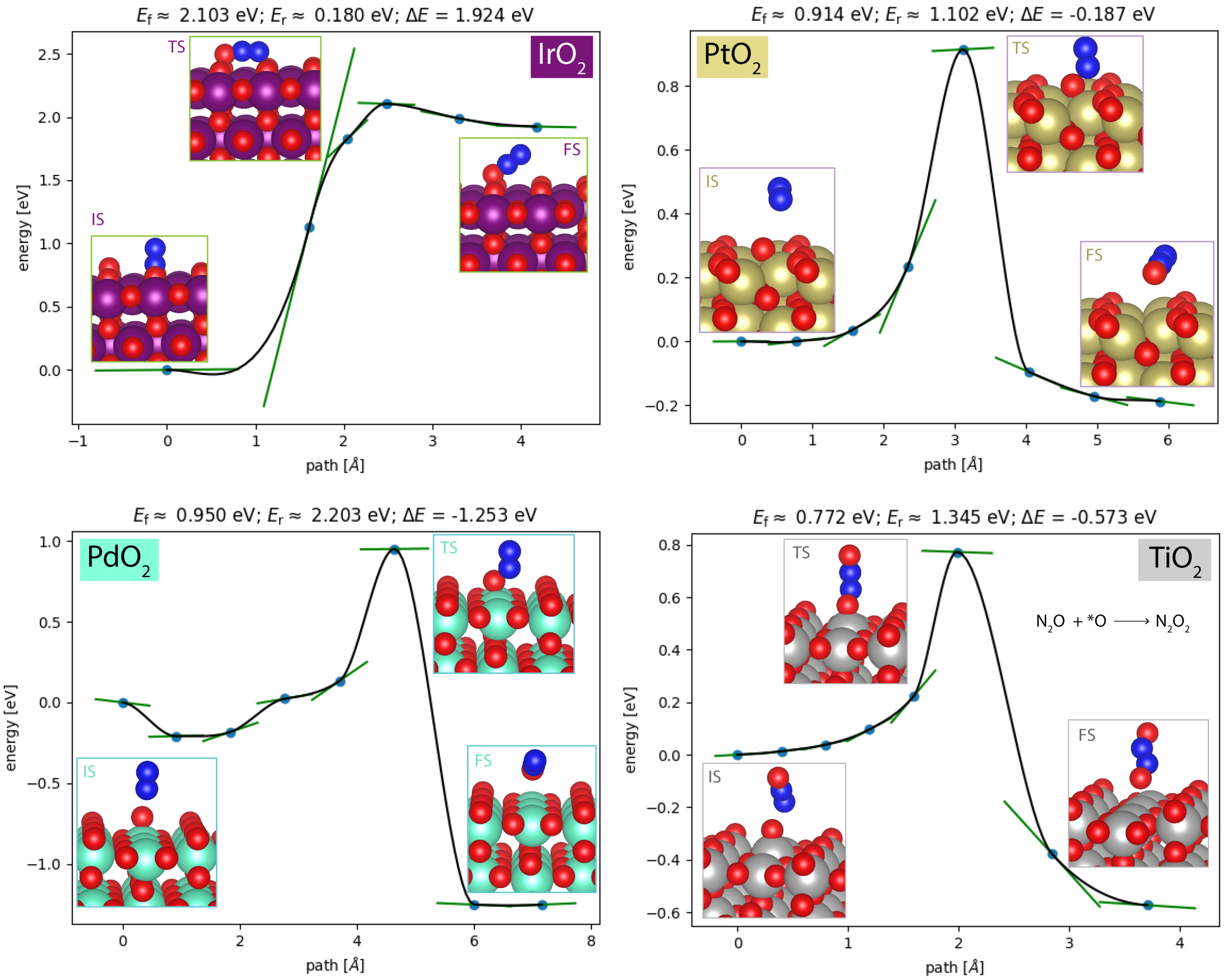

S7 Influence of solvation on N_2OR binding energies

The adsorption of the N_2OR intermediates with explicitly adsorbed water molecules on 5-fold coordinated titanium metal sites were studied. The binding energies on TiO_2 with the inclusion of explicit water molecules are same as that of vacuum (Table S7). Similar observations have been made earlier for OER intermediates on rutile oxides by others.[57]

RPBE exchange-correlation (xc) functional was used for all of the calculations.[35] For

the solvation analysis we have considered a slab modeled by a periodically repeated 2 × 2 unit cell in the x- and y-directions and a four layer thick slab. The two topmost layers were allowed to relax while the bottom two layers are constrained. The slab is aligned in the center with 10 Å vacuum on each side of the slab in the z-direction. We include the dipole corrections in the direction perpendicular to the slab. Considering a thick slab helps overcome the limitations of RPBE in underestimating the water stability due to the lack of van der Waals forces.[58] The k-point sampling consisted of Γ-centered [4 × 2 × 1] grid. We first investigated the adsorption of water molecules on the 5-coordinated Ti atom site. Different configurations were taken into account and the most stable form is considered for further calculations. The adsorption of the first water molecules via the oxygen to the 5-fold coordinated Ti sites can be seen in Figure S14. The water molecule adsorb on the surface without dissociating. The average water adsorption energy on $TiO_2(110)$ is -0.44 eV.

Figure S14: 0.0 ML and 0.5 ML water coverages on the defect-free $TiO_2(110)$ surface. (ML = monolayer)

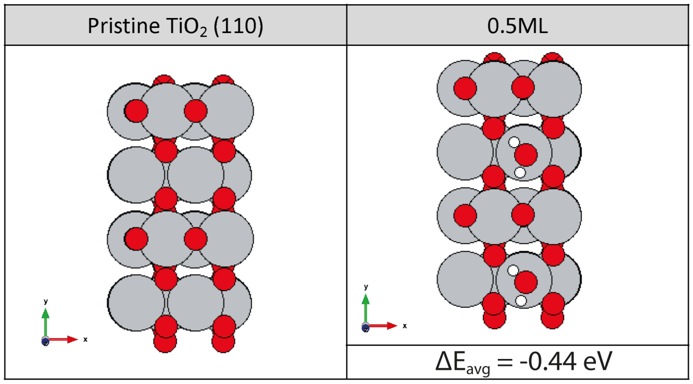

The next step was to identify if the presence of explicitly adsorbed water molecules have any impact on the binding energies of the N_2OR intermediates. Figure S15 shows adsorption energies of the OER intermediates, i.e., HO* and O* in the presence of N_2 as a function of total coverage (adsorbate coverage + water coverage). We find that the presence of explicitly adsorbed H_2O has very small impact on the intermediates adsorption energy. Thus, the solvation effects can be safely ignored while calculating the free energies of the

N_2OR intermediates considered in this work.

Figure S15: Calculated total energy of the intermediates in the presence of N_2 for the defect-free $TiO_2(110)$ surface.

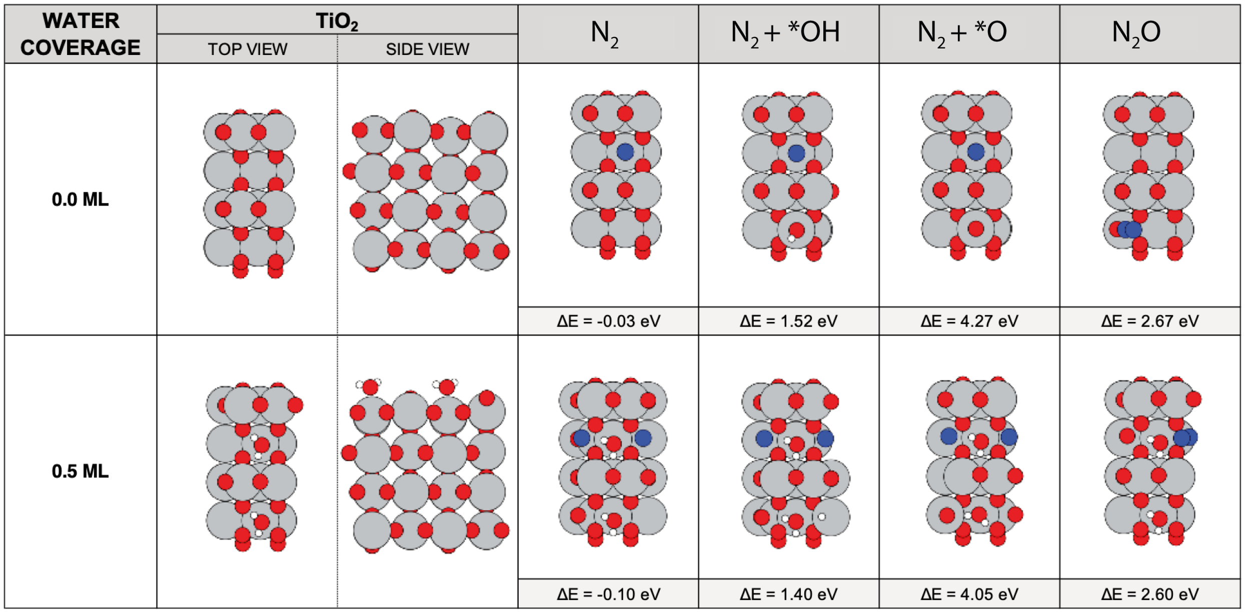

S8 N_2OR on high coverage TiO_2 surface

We found that the dipole corrections do not seem to change the binding energies much since sufficient vacuum was applied in the z-direction. Therefore for saving computing time all the computations in this section were done without any dipole corrections. Similar to the low coverage case, we exhaustively considered all possible binding modes/conformations of each of the N_2OR adsorbate on high $*O/*OH$-covered TiO_2 surface as well. From figure S16-S21, we show the DFT converged structures. The binding energies of N_2OR adsorbates do not change much on high $*O/*OH$-covered TiO_2 surface. The N_2OR adsorbates mostly combine with the surface $*O$ or $*OH$ to form a wide variety of stable species on the surface (Figure S17, S18, S20 and S21).

In the figure S16, we considered OH^* and O^* covered TiO_2 surfaces for N_2OR. Increasing the O/OH-coverage of the surface does not seem to influence the binding of N_2OH (Figure

Figure S16: Different binding modes and conformations of N_2OH on high coverage $TiO_2(110)$ surface. The numbers are the binding energies (no free energy correction) of the adsorbates in eV.

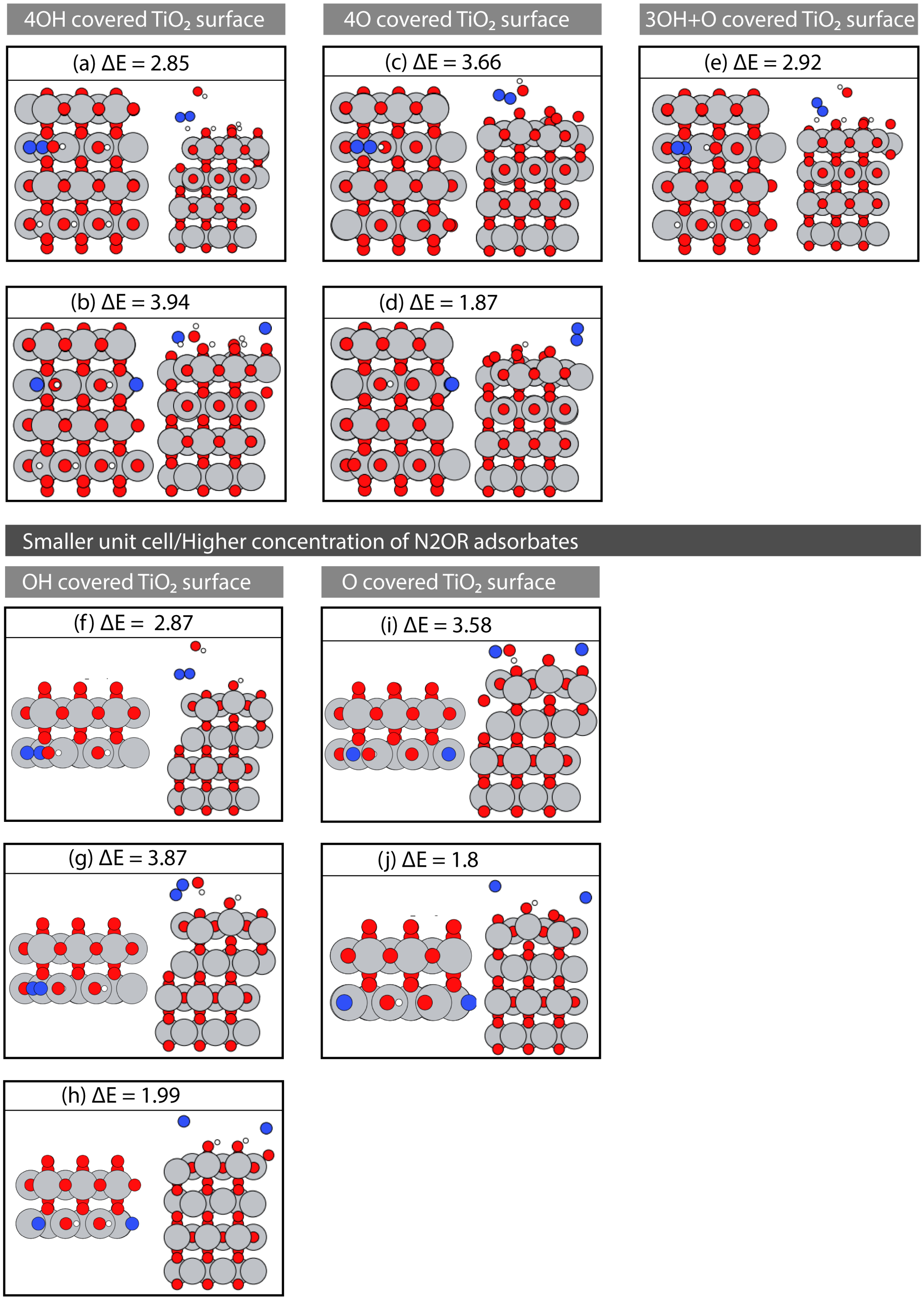

S16(a-c, e)). As indicated by S16(d), the binding of OER intermediate OH^* in favorable over the formation of N_2OH even on oxygen-rich surface. We used a smaller unit cell S16(f-j) to analyze the influence of concentration of N_2OH adsorbates on its binding. The binding of N_2OH is almost unchanged with the smaller unit cell. The presence of OH^* and O^* on the surface enables N_2OH to form other stable species like $N_2O + H_2O$ or $N_2O + OH$ or $HONNO$ as shown in the figure S17. All of these emphasize that TiO_2 is a poor N_2OR catalyst.

Figure S17: N_2OH adsorbates combine with the surface oxygen adsorbates to form other structures such as N_2O or HONNO. We show in this figure some of the structures that were obtained using DFT while trying to optimize N_2OH on high coverage TiO_2 surface. The numbers are the binding energies (no free energy correction) of the adsorbates in eV.

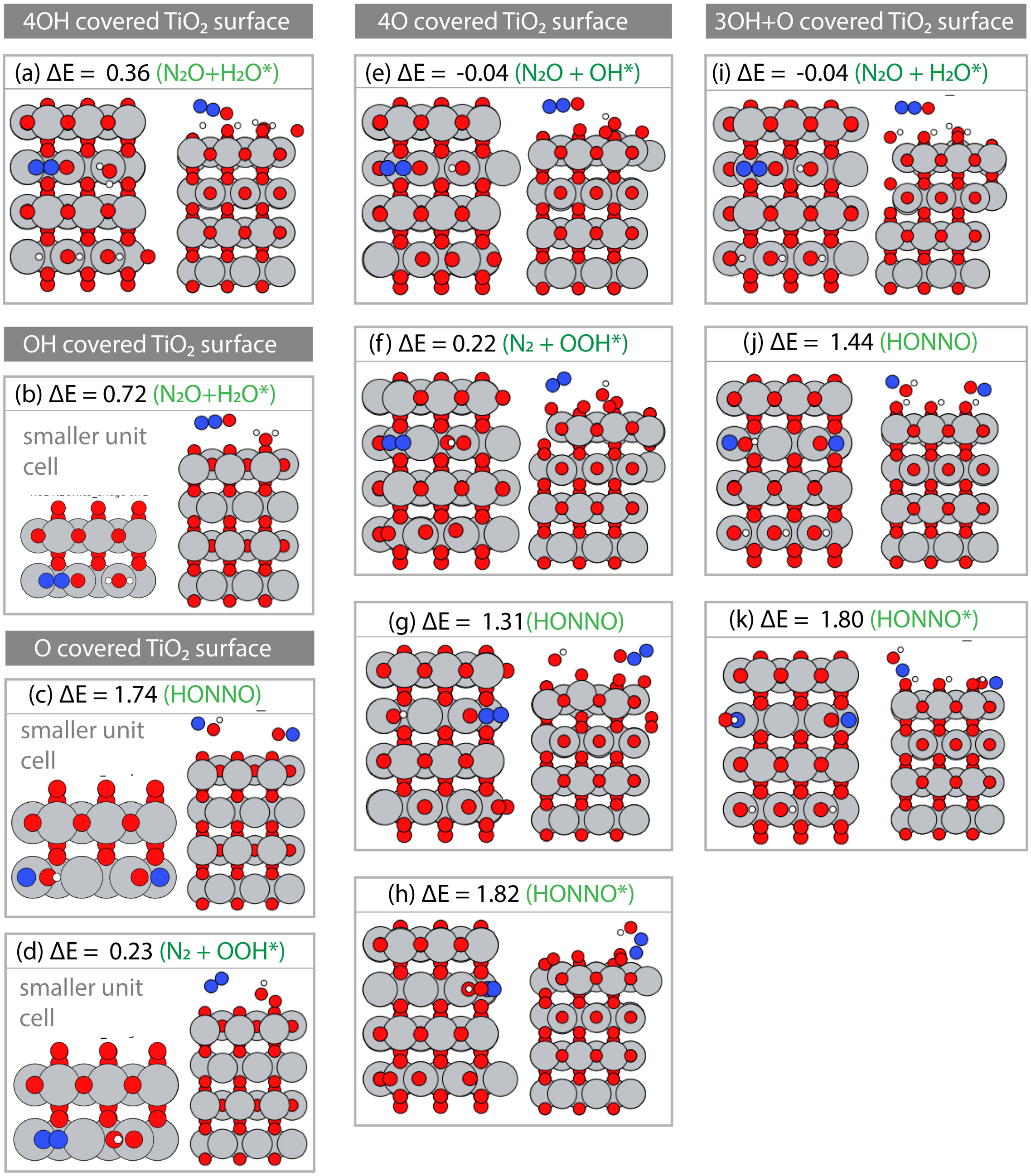

Figure S18: Different binding modes and conformations of N_2O on high coverage catalyst surface. The numbers are the binding energies (no free energy correction) of each of the adsorbates. In image (e) and (i), N_2O abstracts surface oxygen to form N_2O_2.

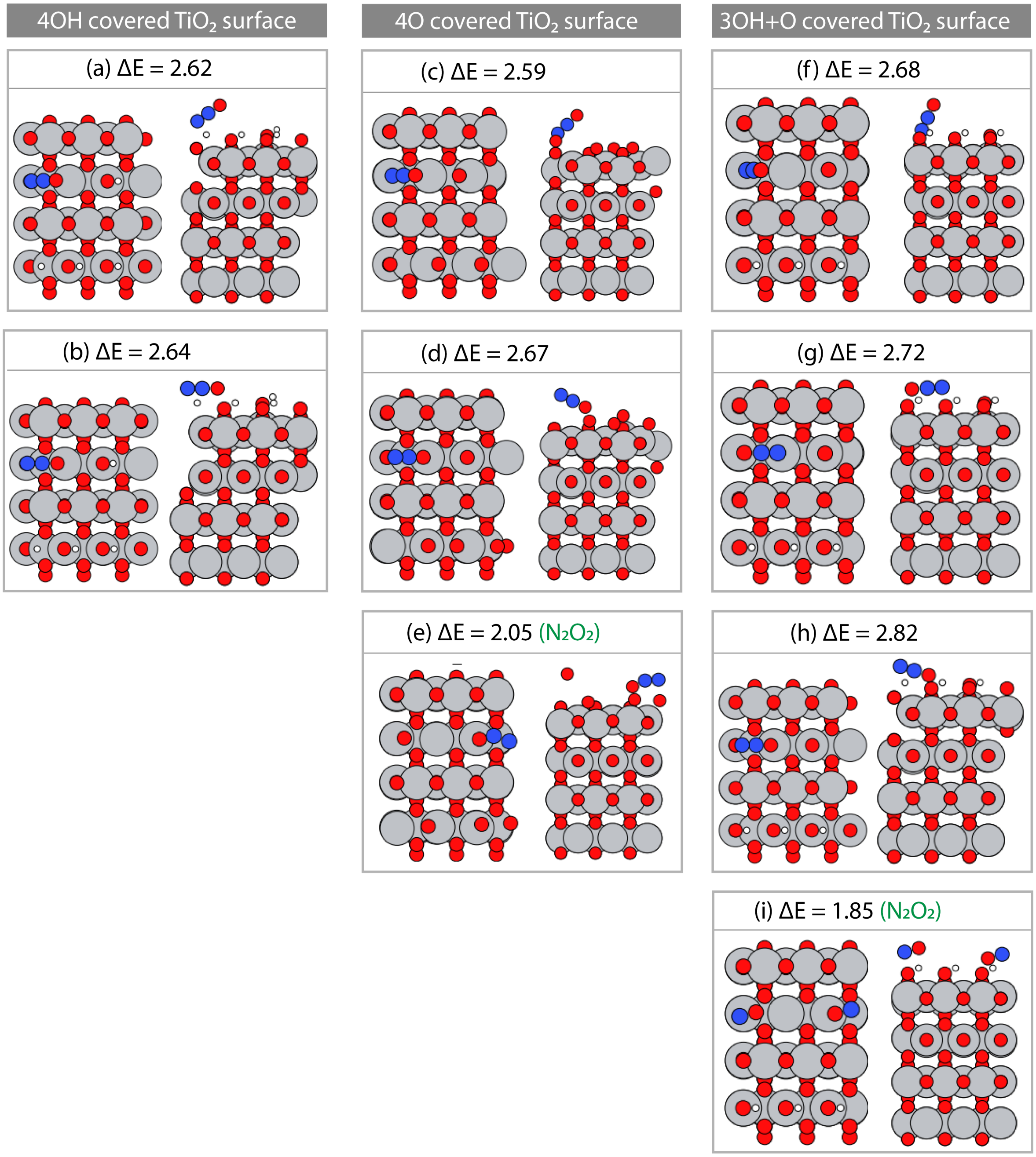

Figure S19: Different binding modes and conformations of HONNO on high coverage catalyst surface. The numbers are the binding energies (no free energy correction) of each of the adsorbates.

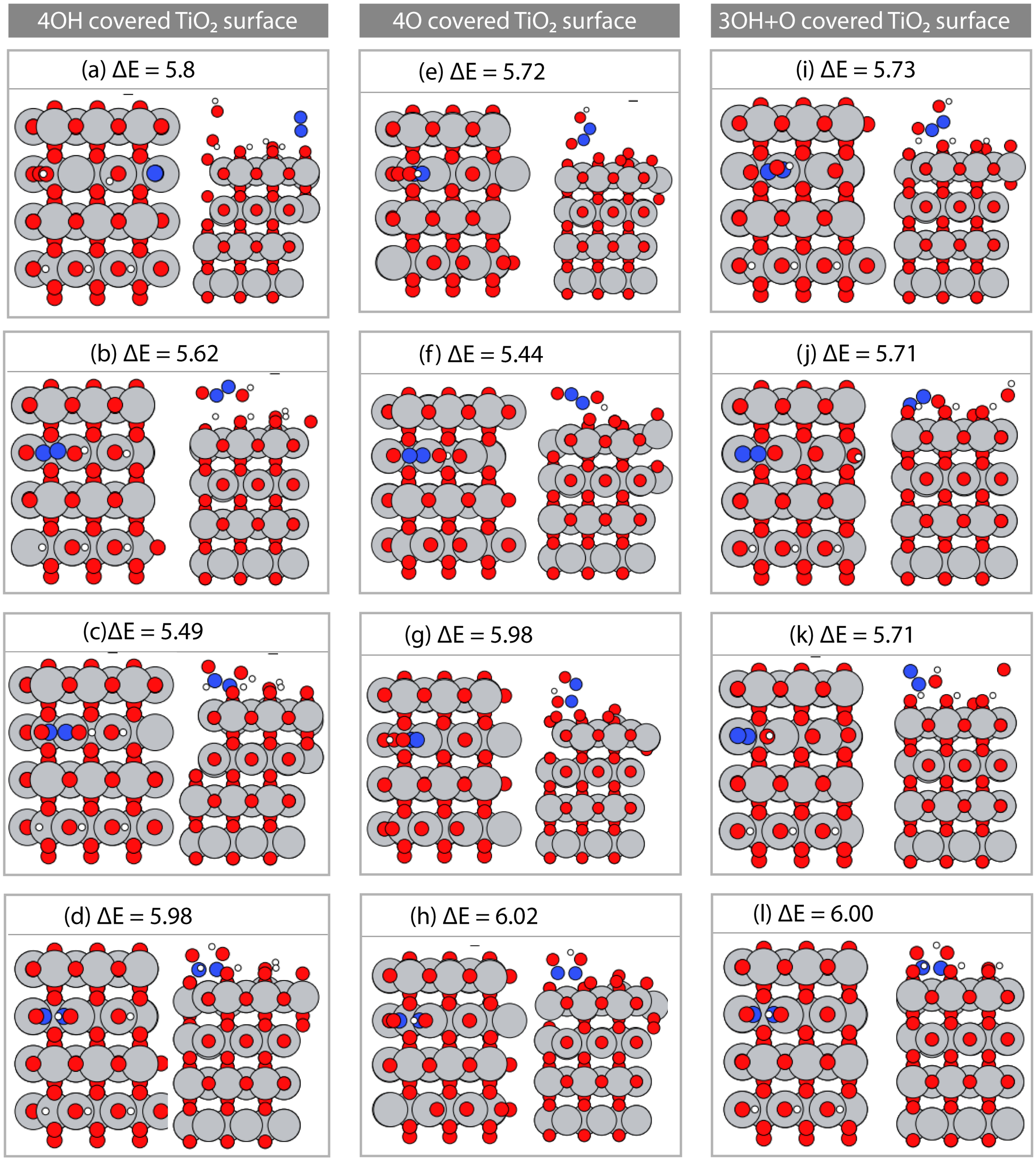

Figure S20: In this figure we show the different species that are formed when HONNO combines with the surface O or OH on high coverage catalyst surface. The numbers are the binding energies (no free energy correction) of each of the adsorbates.

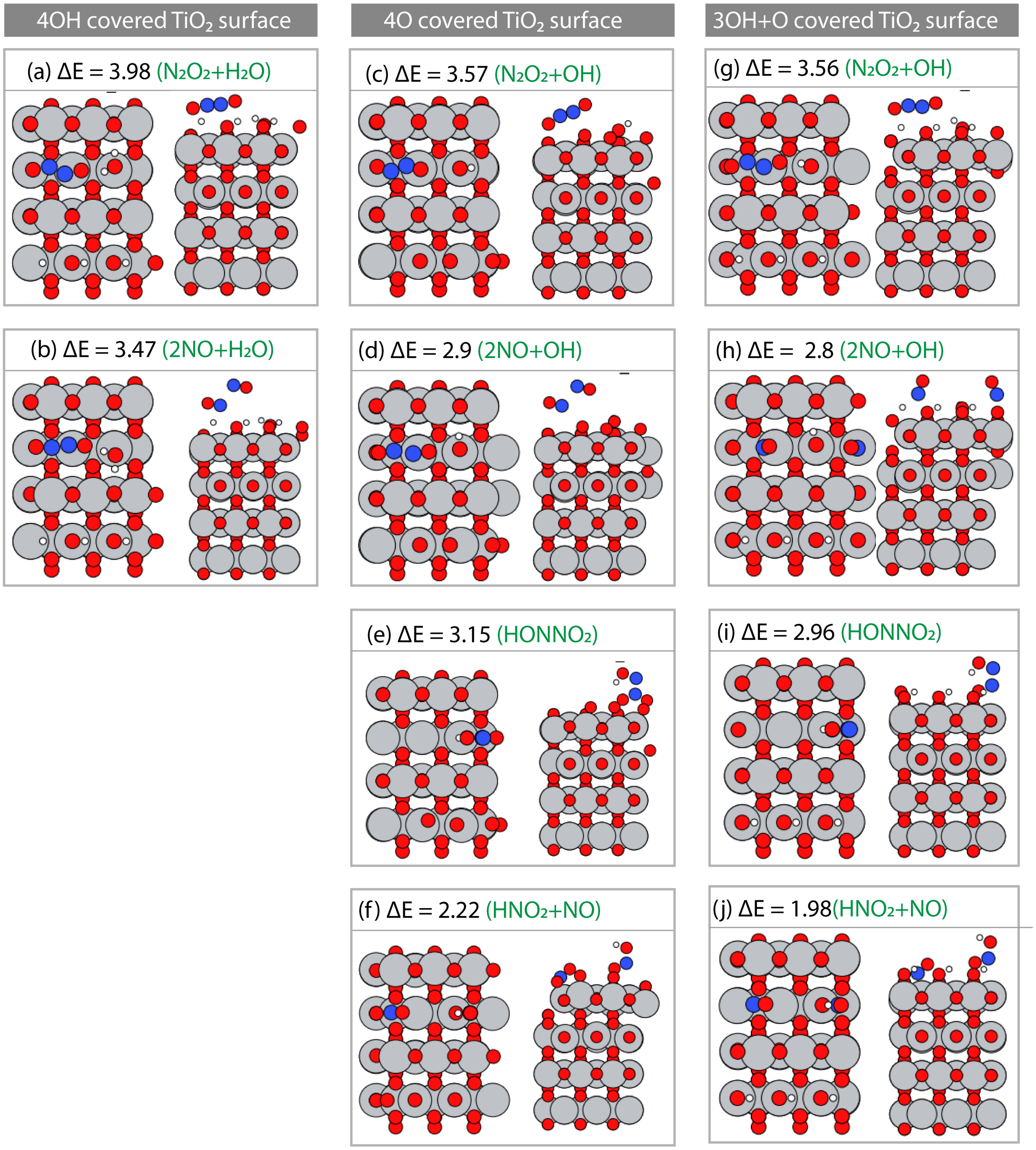

Figure S21: NO adsorbates combine with the surface oxygen to form NO_2 as shown in the figure below. The structures below are obtained using DFT while trying to converge NO on a O/OH-rich TiO_2 surface. The numbers are the binding energies (no free energy correction) of each of the adsorbates.

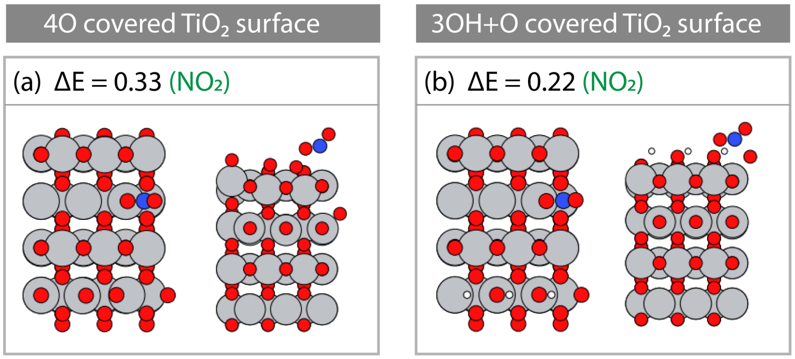

S9 Data for molecules

S9.1 Gaussian 09 SMD(H_2O)/B3LYP-D3/def2tzvp data

NImag refers to the number of imaginary frequencies. 'sol' refers to the SMD(H_2O)/B3LYP-D3/def2tzvp data while 'gaseous' refers to the B3LYP-D3/def2tzvp data. 'sol_from_gaseous' refers to the B3LYP-D3/def2tzvp data with thermochemistry at 0.0313 atm and standard temperature.

```
N2OH(gaseous)

------------------------------------------------------------------------

NImag = 0


 Zero-point correction=                           0.015075 (Hartree/Particle)

 Thermal correction to Energy=                    0.020722

 Thermal correction to Enthalpy=                  0.021666

 Thermal correction to Gibbs Free Energy=        -0.011827

 Sum of electronic and zero-point Energies=           -185.328918

 Sum of electronic and thermal Energies=              -185.323271
```

Sum of electronic and thermal Enthalpies= -185.322327

Sum of electronic and thermal Free Energies= -185.355820

8	0.070326	-2.377590	0.000000
1	0.105521	-1.401271	0.000000
7	0.000000	0.915691	0.000000
7	-0.095447	2.001735	-0.000000

N2OH(sol)

NImag = 0

Zero-point correction= 0.015511 (Hartree/Particle)

Thermal correction to Energy= 0.020873

Thermal correction to Enthalpy= 0.021817

Thermal correction to Gibbs Free Energy= -0.012331

Sum of electronic and zero-point Energies= -185.336271

Sum of electronic and thermal Energies= -185.330910

Sum of electronic and thermal Enthalpies= -185.329965

Sum of electronic and thermal Free Energies= -185.364113

1	1.541257	0.343426	0.000016

7	-0.968792	0.136473	-0.000004
7	-2.029944	-0.113798	0.000003
8	2.431237	-0.062768	-0.000001

N2O(gaseous)

NImag = 0

```
 Zero-point correction=                           0.011199 (Hartree/Particle)
 Thermal correction to Energy=                    0.013867
 Thermal correction to Enthalpy=                  0.014811
 Thermal correction to Gibbs Free Energy=        -0.010094
 Sum of electronic and zero-point Energies=           -184.732796
 Sum of electronic and thermal Energies=              -184.730128
 Sum of electronic and thermal Enthalpies=            -184.729183
 Sum of electronic and thermal Free Energies=         -184.754089
```

7	-0.000000	-0.000000	0.073429
7	-0.000000	-0.000000	1.194557
8	0.000000	0.000000	-1.109488

N2O(sol)

```
NImag = 0

 Zero-point correction=                           0.011103 (Hartree/Particle)
 Thermal correction to Energy=                    0.013774
 Thermal correction to Enthalpy=                  0.014719
 Thermal correction to Gibbs Free Energy=        -0.010190
 Sum of electronic and zero-point Energies=           -184.732259
 Sum of electronic and thermal Energies=              -184.729587
 Sum of electronic and thermal Enthalpies=            -184.728643
 Sum of electronic and thermal Free Energies=         -184.753552


      7              0.000000   -0.000000    0.074201
      7              0.000000   -0.000000    1.193337
      8             -0.000000    0.000000   -1.109097
-------------------------------------------------------------------------

HONNO(gaseous)
-------------------------------------------------------------------------
NImag = 0

 Zero-point correction=                           0.024655 (Hartree/Particle)
 Thermal correction to Energy=                    0.028795
 Thermal correction to Enthalpy=                  0.029740
 Thermal correction to Gibbs Free Energy=        -0.001785
 Sum of electronic and zero-point Energies=           -260.476842
```

```
 Sum of electronic and thermal Energies=              -260.472702
 Sum of electronic and thermal Enthalpies=            -260.471758
 Sum of electronic and thermal Free Energies=         -260.503282
```

```
      8           -0.715825    1.592738    0.000000
      7            0.000000    0.616155    0.000000
      7           -0.283847   -0.584184    0.000000
      8            0.895389   -1.340358    0.000000
      1            0.550418   -2.242832    0.000000
------------------------------------------------------------------------
```

```
HONNO(sol)
------------------------------------------------------------------------
NImag = 0

 Zero-point correction=                           0.024469 (Hartree/Particle)
 Thermal correction to Energy=                    0.028591
 Thermal correction to Enthalpy=                  0.029535
 Thermal correction to Gibbs Free Energy=        -0.001935
 Sum of electronic and zero-point Energies=           -260.480685
 Sum of electronic and thermal Energies=              -260.476564
 Sum of electronic and thermal Enthalpies=            -260.475620
 Sum of electronic and thermal Free Energies=         -260.507089
```

8	-0.730194	1.590543	0.000000
7	0.000000	0.617715	-0.000000
7	-0.271140	-0.583703	0.000000
8	0.896979	-1.338994	-0.000000
1	0.563702	-2.250476	-0.000000

NO(gaseous)

NImag = 0

```
 Zero-point correction=                           0.004503 (Hartree/Particle)
 Thermal correction to Energy=                    0.006864
 Thermal correction to Enthalpy=                  0.007809
 Thermal correction to Gibbs Free Energy=        -0.015480
 Sum of electronic and zero-point Energies=           -129.942729
 Sum of electronic and thermal Energies=              -129.940368
 Sum of electronic and thermal Enthalpies=            -129.939424
 Sum of electronic and thermal Free Energies=         -129.962712
```

7	0.000000	0.000000	-0.610631
8	0.000000	0.000000	0.534302

```
NO(sol)
-----------------------------------------------------------------------
NImag = 0

 Zero-point correction=                           0.004493 (Hartree/Particle)
 Thermal correction to Energy=                    0.006854
 Thermal correction to Enthalpy=                  0.007798
 Thermal correction to Gibbs Free Energy=        -0.015489
 Sum of electronic and zero-point Energies=           -129.940070
 Sum of electronic and thermal Energies=              -129.937708
 Sum of electronic and thermal Enthalpies=            -129.936764
 Sum of electronic and thermal Free Energies=         -129.960051



      7             0.000000    0.000000   -0.610291
      8             0.000000    0.000000    0.534005
-----------------------------------------------------------------------

HNO2(gaseous)
-----------------------------------------------------------------------
NImag = 0

 Zero-point correction=                           0.020121 (Hartree/Particle)
 Thermal correction to Energy=                    0.023361
 Thermal correction to Enthalpy=                  0.024305
 Thermal correction to Gibbs Free Energy=        -0.003879
```

```
 Sum of electronic and zero-point Energies=          -205.776024
 Sum of electronic and thermal Energies=             -205.772785
 Sum of electronic and thermal Enthalpies=           -205.771841
 Sum of electronic and thermal Free Energies=        -205.800024
```

```
     7             0.000000    0.518074    0.000000
     8            -1.109947    0.171336    0.000000
     8             0.889553   -0.602083    0.000000
     1             1.763148   -0.180551    0.000000
-------------------------------------------------------------------------
```

HNO2(sol)

```
-------------------------------------------------------------------------
NImag = 0

 Zero-point correction=                           0.020101 (Hartree/Particle)
 Thermal correction to Energy=                    0.023299
 Thermal correction to Enthalpy=                  0.024244
 Thermal correction to Gibbs Free Energy=        -0.003856
 Sum of electronic and zero-point Energies=          -205.779939
 Sum of electronic and thermal Energies=             -205.776741
 Sum of electronic and thermal Enthalpies=           -205.775797
 Sum of electronic and thermal Free Energies=        -205.803897
```

```
     7              0.000000    0.510031    0.000000
     8             -1.113638    0.135102    0.000000
     8              0.892049   -0.563092    0.000000
     1              1.772711   -0.146295    0.000000
-------------------------------------------------------------------------
```

NO2(gaseous)

```
-------------------------------------------------------------------------
NImag = 0

 Zero-point correction=                           0.008787 (Hartree/Particle)
 Thermal correction to Energy=                    0.011718
 Thermal correction to Enthalpy=                  0.012662
 Thermal correction to Gibbs Free Energy=        -0.014559
 Sum of electronic and zero-point Energies=           -205.158462
 Sum of electronic and thermal Energies=              -205.155531
 Sum of electronic and thermal Enthalpies=            -205.154587
 Sum of electronic and thermal Free Energies=         -205.181808


     7              0.000000    0.000000    0.320705
     8              0.000000    1.097888   -0.140308
     8             -0.000000   -1.097888   -0.140308
-------------------------------------------------------------------------
```

NO2(sol)

```
--------------------------------------------------------------------------
NImag = 0

 Zero-point correction=                           0.008628 (Hartree/Particle)
 Thermal correction to Energy=                    0.011561
 Thermal correction to Enthalpy=                  0.012505
 Thermal correction to Gibbs Free Energy=        -0.014721
 Sum of electronic and zero-point Energies=           -205.153621
 Sum of electronic and thermal Energies=              -205.150688
 Sum of electronic and thermal Enthalpies=            -205.149744
 Sum of electronic and thermal Free Energies=         -205.176970


     7             -0.000000   -0.000000    0.321848
     8              0.000000    1.096979   -0.140809
     8             -0.000000   -1.096979   -0.140809
--------------------------------------------------------------------------
```

HNO3(gaseous)

```
--------------------------------------------------------------------------
NImag = 0

 Zero-point correction=                           0.026242 (Hartree/Particle)
 Thermal correction to Energy=                    0.029778
 Thermal correction to Enthalpy=                  0.030722
```

```
 Thermal correction to Gibbs Free Energy=         0.000495
 Sum of electronic and zero-point Energies=           -280.989022
 Sum of electronic and thermal Energies=              -280.985486
 Sum of electronic and thermal Enthalpies=            -280.984542
 Sum of electronic and thermal Free Energies=         -281.014768
```

7	0.000000	0.155512	0.000000
8	-0.980725	0.832152	0.000000
8	-0.263403	-1.232118	0.000000
8	1.166026	0.467551	0.000000
1	0.624814	-1.629261	0.000000

HNO3(sol)

NImag = 0

```
 Zero-point correction=                           0.026029 (Hartree/Particle)
 Thermal correction to Energy=                    0.029525
 Thermal correction to Enthalpy=                  0.030469
 Thermal correction to Gibbs Free Energy=         0.000315
 Sum of electronic and zero-point Energies=           -280.993665
 Sum of electronic and thermal Energies=              -280.990168
 Sum of electronic and thermal Enthalpies=            -280.989224
 Sum of electronic and thermal Free Energies=         -281.019379
```

7	0.000000	0.136569	0.000000
8	-1.001736	0.802684	0.000000
8	-0.230420	-1.223750	0.000000
8	1.150425	0.506784	0.000000
1	0.653844	-1.641725	0.000000

H2(gaseous)

NImag = 0

```
 Zero-point correction=                           0.010074 (Hartree/Particle)
 Thermal correction to Energy=                    0.012435
 Thermal correction to Enthalpy=                  0.013379
 Thermal correction to Gibbs Free Energy=        -0.001416
 Sum of electronic and zero-point Energies=             -1.169575
 Sum of electronic and thermal Energies=                -1.167214
 Sum of electronic and thermal Enthalpies=              -1.166270
 Sum of electronic and thermal Free Energies=           -1.181065
```

1	0.000000	0.000000	0.371966
1	0.000000	0.000000	-0.371966

```
--------------------------------------------------------------------------

H2O(sol_from_gaseous)
--------------------------------------------------------------------------
NImag = 0

 Zero-point correction=                           0.021170 (Hartree/Particle)
 Thermal correction to Energy=                    0.024006
 Thermal correction to Enthalpy=                  0.024950
 Thermal correction to Gibbs Free Energy=         0.000256
 Sum of electronic and zero-point Energies=           -76.441834
 Sum of electronic and thermal Energies=              -76.438998
 Sum of electronic and thermal Enthalpies=            -76.438054
 Sum of electronic and thermal Free Energies=         -76.462748


      8              0.000000    0.000000    0.116877
      1             -0.000000    0.765097   -0.467508
      1             -0.000000   -0.765097   -0.467508
--------------------------------------------------------------------------


N2(gaseous)
--------------------------------------------------------------------------
NImag = 0 (lowest freq = 2452.8811 cm-1)
```

```
 Zero-point correction=                           0.005588 (Hartree/Particle)
 Thermal correction to Energy=                    0.007949
 Thermal correction to Enthalpy=                  0.008893
 Thermal correction to Gibbs Free Energy=        -0.012837
 Sum of electronic and zero-point Energies=           -109.567674
 Sum of electronic and thermal Energies=              -109.565313
 Sum of electronic and thermal Enthalpies=            -109.564369
 Sum of electronic and thermal Free Energies=         -109.586099
```

```
     7              0.000000    0.000000    0.545454
     7              0.000000    0.000000   -0.545454
-----------------------------------------------------------------------
```

S9.2 VASP data

The converged ASE trajectory file of all the systems discussed in the main text are provided in the zipped file.

Table S12: DFT energies of OER adsorbates on different oxides obtained using VASP.

Structure	E_{DFT}
TiO_2	-1251.681
TiO_2−OH	-1261.412
TiO_2−O	-1255.2
TiO_2−OOH	-1266.142
5 O−TiO_2	-1267.473
5 O−TiO_2−OH	-1277.004
5 O−TiO_2−O	-1270.14
5 O−TiO_2−OOH	-1281.952
5 OH−TiO_2	-1298.096
5 OH−TiO_2−OH	-1307.019
5 OH−TiO_2−O	-1301.072
5 OH−TiO_2−OOH	-1312.181
PdO_2	-738.7936
PdO_2−OH	-748.3524
PdO_2−O	-742.505
PdO_2−OOH	-753.2379
PtO_2	-534.7324
PtO_2−OH	-545.1381
PtO_2−O	-539.4837
PtO_2−OOH	-549.7647
IrO_2	-986.3466
IrO_2−OH	-997.6132
IrO_2−O	-992.5554
IrO_2−OOH	-1002.238
5 O−IrO_2	-1017.211
5 O−IrO_2−OH	-1028.372
5 O−IrO_2−O	-1023.336
5 O−IrO_2−OOH	-1032.967

Table S13: DFT single point energies of different TiO_2 surfaces at varying electric field ($\vec{E}$) obtained using quantum espresso. The computational details are discussed in the section S2.5. The $O-TiO_2$ and N_2+TiO_2 correspond to the initial structure ($N_2+O-TiO_2$) with N_2 and $*O$ removed from the surface, respectively.

$\vec{E}$ (V/Å)	$E(TiO_2)$	$E(O-TiO_2)$	$E(N_2+O-TiO_2)$	$E(N_2+TiO_2)$	$E(N_2\cdots O\cdots TiO_2)$
-1	-132726.1378	-133292.4153	-133842.9547	-133273.9177	-133841.655
-0.8	-132724.6389	-133290.7525	-133841.219	-133272.1873	-133839.9797
-0.6	-132723.6041	-133289.4343	-133839.8549	-133270.83	-133838.6803
-0.4	-132722.8801	-133288.3804	-133838.8438	-133270.1398	-133837.5583
-0.2	-132722.4615	-133287.8313	-133838.1837	-133269.8936	-133837.1658
0	-132722.3465	-133287.5468	-133837.8598	-133269.8375	-133836.9566
0.2	-132722.5357	-133287.6073	-133837.917	-133270.1234	-133837.1135
0.4	-132723.0299	-133288.0123	-133838.3111	-133270.7514	-133837.6365
0.6	-132723.8294	-133288.7626	-133839.0596	-133271.7207	-133838.5253
0.8	-132724.9335	-133289.857	-133840.1874	-133273.0307	-133839.7791
1	-132726.3412	-133291.2946	-133841.705	-133274.6781	-133841.3957

Table S14: ΔE calculated from the DFT energies in table S13 with respect to the pristine surface. Section S2.5 describes how ΔE is obtaned from the DFT energies in table S13. We use these ΔE in the main text for figure 4(b).

$\vec{E}$ (V/Å)	$\Delta E(TiO_2)$	$\Delta E(O-TiO_2)$	$\Delta E(N_2+O-TiO_2)$	$\Delta E(N_2+TiO_2)$	$\Delta E(N_2\cdots O\cdots TiO_2)$
-1	0	-1.08	-1.30	-0.29	-0.91
-0.8	0	-0.91	-1.07	-0.06	-0.73
-0.6	0	-0.63	-0.74	0.27	-0.47
-0.4	0	-0.30	-0.45	0.23	-0.07
-0.2	0	-0.17	-0.21	0.06	-0.09
0	0	0.00	0.00	0.00	0.00
0.2	0	0.13	0.13	-0.10	0.03
0.4	0	0.22	0.23	-0.23	0.00
0.6	0	0.27	0.28	-0.40	-0.09
0.8	0	0.28	0.26	-0.61	-0.24
1	0	0.25	0.15	-0.85	-0.44

Table S15: DFT energies and binding energies of 0.0 monolayer structures discussed in figure S15.

Structures	E_{DFT}	ΔE
TiO_2	-833.8084184	
TiO_2-N_2	-851.034485	-0.03
$TiO_2-N_2{}^{+}{*}OH$	-860.6532677	1.52
$TiO_2-N_2{}^{+}{*}O$	-854.3844729	4.27
TiO_2-N_2O	-855.9895896	2.67

Table S16: DFT energies and binding energies of 0.5 monolayer structures discussed in figure S15.

Structures	E_{DFT}	ΔE
TiO_2	-864.07175817	
TiO_2-N_2	-881.37601941	-0.10
$TiO_2-N_2{}^{+}{*}OH$	-891.03887041	1.40
$TiO_2-N_2{}^{+}{*}O$	-884.86290761	4.05
TiO_2-N_2O	-886.3136465	2.60

Table S17: DFT energies of TiO_2 in different unit cell sizes at varying coverages. These electronic energies computed without any dipole corrections are used as references for the high coverage N_2OR computations.

Structure	E_{DFT}	ΔE
$4\,OH-TiO_2$ (3x2)	-1289.009991	0
$4\,O-TiO_2$ (3x2)	-1264.697815	0
$3\,OH + O-TiO_2$ (3x2)	-1282.7985	0
$OH-TiO_2$ (3x1)	-635.435812	0
$O-TiO_2$ (3x1)	-629.187711	0

Table S18: DFT energies and binding energies of structures discussed in figure S16.

Structure	E_{DFT}	ΔE
S16-a	-1314.5276	2.85
S16-b	-1313.43469	3.94
S16-c	-1289.404593	3.66
S16-d	-1291.198617	1.87
S16-e	-1308.241033	2.92
S16-f	-660.935746	2.87
S16-g	-659.928956	3.87
S16-h	-661.810512	1.99
S16-i	-653.970917	3.58
S16-j	-655.75939	1.8

Table S19: DFT energies and binding energies of strcutures discussed in figure S17

Structure	E_{DFT}	ΔE
S17-a	-1317.018884	0.36
S17-b	-663.086371	0.72
S17-c	-655.811487	1.74
S17-d	-657.321496	0.23
S17-e	-1293.10027	-0.04
S17-f	-1292.84796	0.22
S17-g	-1291.750752	1.31
S17-h	-1291.240565	1.82
S17-i	-1311.2031	-0.04
S17-j	-1309.730246	1.44
S17-k	-1309.363667	1.8

Table S20: DFT energies and binding energies of strcutures discussed in figure S18

Structure	E_{DFT}	ΔE
S18-a	-1311.230348	2.62
S18-b	-1311.216896	2.64
S18-c	-1286.949007	2.59
S18-d	-1286.873383	2.67
S18-e	-1287.494633	2.05
S18-f	-1304.958425	2.68
S18-g	-1304.921375	2.72
S18-h	-1304.820813	2.82
S18-i	-1305.794129	1.85

Table S21: DFT energies and binding energies of strcutures discussed in figure S19

Structure	E_{DFT}	ΔE
S19-a	-1319.217749	5.8
S19-b	-1319.405402	5.62
S19-c	-1319.535897	5.49
S19-d	-1319.04044	5.98
S19-e	-1294.99102	5.72
S19-f	-1295.267324	5.44
S19-g	-1294.73107	5.98
S19-h	-1294.686626	6.02
S19-i	-1313.082726	5.73
S19-j	-1313.096291	5.71
S19-k	-1313.10349	5.71
S19-l	-1312.806167	6

Table S22: DFT energies and binding energies of strcutures discussed in figure S20

Structure	E_{DFT}	ΔE
S20-a	-1321.038494	3.98
S20-b	-1321.547434	3.47
S20-c	-1297.142127	3.57
S20-d	-1297.809515	2.9
S20-e	-1297.563355	3.15
S20-f	-1298.492787	2.22
S20-g	-1315.246226	3.56
S20-h	-1316.007232	2.8
S20-i	-1315.85025	2.96
S20-j	-1316.833184	1.98

Table S23: DFT energies and binding energies of strcutures discussed in figure S21

Structure	E_{DFT}	ΔE
S21-a	-1280.609901	0.33
S21-b	-1298.823522	0.22

References

(1) Du, S.; Francisco, J. S. OH.N2 and SH.N2 radical-molecule van der Waals complex. *J. Chem. Phys.* **2009**, *131*, 064307.

(2) Ikabata, Y.; Nakai, H. Assessment of local response dispersion method for open-shell systems. *Chem. Phys. Lett.* **2013**, *556*, 386–392.

(3) Bozzelli, J. W.; Dean, A. M. O + NNH: A Possible New Route for NOx Formation in Flames. *Int. J. Chem. Kinet.* **1995**, *27*, 1097–1109.

(4) Bradley, K. S.; Mccabe, P.; Schatz, G. C.; Walch, S. P. A theoretical study of the NH + NO reaction. *J. Chem. Phys.* **1995**, *6696*.

(5) Castillo, J. F.; Collins, M. A.; Aoiz, F. J.; Bañares, L. Quasiclassical trajectory study of the dynamics of the H+N2O reaction on a new potential energy surface. *J. Chem. Phys* **2003**, *118*, 7303.

(6) Teffo, J. L.; Chédin, A. Internuclear potential and equilibrium structure of the nitrous oxide molecule from rovibrational data. *J. Mol. Spectrosc.* **1989**, *135*, 389–409.

(7) Wang, F.; Harcourt, R. D. Electronic Structure Study of the N2O Isomers Using Post-Hartree-Fock and Density Functional Theory Calculations. *J. Phys. Chem. A* **2000**, *104*, 1304–1310.

(8) Crusius, J.-P.; Hellmann, R.; Hassel, E.; Bich, E. Ab initio intermolecular potential energy surface and thermophysical properties of nitrous oxide. *J. Chem. Phys.* **2015**, *142*, 244307.

(9) Aplincourt, P.; Bohr, F.; Ruiz-Lopez, M. F. Density functional studies of compounds involved in atmospheric chemistry: nitrogen oxides. *J. Mol. Struct.* **1998**, *1280*.

(10) Kazazić, S.; Kazazić, S. P.; Klasinc, L.; McGlynn, S. P.; Pryor, W. A. Proton affinities of N–O anions and their protonated forms. *J. Phys. Org. Chem.* **2002**, *15*, 728–731.

(11) Chakraborty, D.; Hsu, C.-C.; Lin, M. C. Theoretical studies of nitroamino radical reactions: Rate constants for the unimolecular decomposition of HNNO2 and related bimolecular processes. *J. Chem. Phys.* **1998**, *109*, 8887–8896.

(12) Fedoseev, G.; Ioppolo, S.; Lamberts, T.; Zhen, J. F.; Cuppen, H. M.; Fedoseev, G.; Ioppolo, S.; Lamberts, T.; Zhen, J. F.; Cuppen, H. M. Efficient surface formation route of interstellar hydroxylamine through NO hydrogenation. II. The multilayer regime in interstellar relevant ices. *J. Chem. Phys.* **2012**, *137*, 054714.

(13) Diau, E. W.; Halbgewachs, M. J.; Smith, A. R.; Lin, M. C. Thermal Reduction of NO by H2: Kinetic Measurement and Computer Modeling of the HNO + NO Reaction. *Int. J. Chem. Kinet.* **1995**, *27*, 867–881.

(14) Kirsch, M.; De Groot, H. Formation of peroxynitrite from reaction of nitroxyl anion with molecular oxygen. *J. Biol. Chem.* **2002**, *277*, 13379–13388.

(15) Seddon, W. A.; Fletcher, J. W.; Sopchyshyn, F. C. Pulse Radiolysis of Nitric Oxide in Aqueous Solution. *Can. J. Chem.* **1973**, *51*, 1123–1130.

(16) Poskrebyshev, G. A.; Shafirovich, V.; Lymar, S. V. Disproportionation Pathways of Aqueous Hyponitrite Radicals (HN2O2•/N2O2•-). *J. Phys. Chem. A* **2008**, *112*, 8295–8302.

(17) Francisco, J. S. Protonated nitrous acid (H2ONO+): Molecular structure, vibrational frequencies, and proton affinity. *J. Chem. Phys* **2001**, *115*, 2117.

(18) Glendening, E. D.; Halpern, A. M. Ab initio calculations of nitrogen oxide reactions: Formation of N2O2, N2O3, N2O4, N2O5, and N4O2 from NO, NO2, NO3, and N2O. *J. Chem. Phys.* **2007**, *127*.

(19) Herzberg, G. *Electronic Spectra and Elelctronic Structure of Polyatomic Molecules*; Van Nostrand: New York, 1966.

(20) Burnelle, L.; Beaudouin, P.; Schaad, L. J. Electronic structure of nitrogen dioxide, its ions, and its dimer. *J. Phys. Chem.* **1967**, *71*, 2240–2247.

(21) Rothenberg, S.; Schaefer, H. F. Self-consistent-field wave functions, energies, multipole moments, diamagnetic susceptibility and shielding tensors, and electric field gradient tensors for nitrogen dioxide and ozone. *Mol. Phys.* **1971**, *21*, 317–327.

(22) Fortune, P. J.; Rosenberg, B. J.; England, W. B.; Wahl, A. C. Theoretical studies of atmospheric molecules: SCF and correlated energy levels for the NO2, NO2+ and NO2- systems. *Theor. Chim. Acta* **1977**, *46*, 183–189.

(23) Mota, V. C.; Caridade, P. J. S. B.; Varandas, A. J. C. Toward the Modeling of the NO2(2A") Manifold. *Int. J. Quant. Chem.* **2011**, *111*, 3776–3785.

(24) Fink, W. H. SCF Determination of the Ground and Excited States of NO2. *J. Chem. Phys.* **1968**, *49*, 5054–5060.

(25) Brambila, D. S.; Alex Harvey, a. G.; Houfek, K.; Maš ı, Z.; Smirnova, O. Role of electronic correlations in photoionization of NO2 in the vicinity of the 2A1/2B2 conical intersection. *Phys. Chem. Chem. Phys* **2017**, *19*, 19673–19682.

(26) Nørskov, J. K.; Rossmeisl, J.; Logadottir, A.; Lindqvist, L.; Kitchin, J. R.; Bligaard, T.; Jónsson, H. Origin of the Overpotential for Oxygen Reduction at a Fuel-Cell Cathode. *J. Phys. Chem. B* **2004**, *108*, 17886–17892.

(27) Frisch, M. J. et al. Gaussian 16, Revision A.03; Gaussian, Inc.: Wallingford, CT. 2016.

(28) Grimme, S.; Antony, J.; Ehrlich, S.; Krieg, H. A consistent and accurate ab initio parametrization of density functional dispersion correction (DFT-D) for the 94 elements H-Pu. *J. Chem. Phys.* **2010**, *132*, 154104.

(29) Weigend, F.; Ahlrichs, R. Balanced basis sets of split valence, triple zeta valence and

quadruple zeta valence quality for H to Rn: Design and assessment of accuracy. *Phys. Chem. Chem. Phys.* **2005**, *7*, 3297–3305.

(30) Marenich, A. V.; Cramer, C. J.; Truhlar, D. G. Universal solvation model based on solute electron density and on a continuum model of the solvent defined by the bulk dielectric constant and atomic surface tensions. *J. Phys. Chem. B* **2009**, *113*, 6378–6396.

(31) Hafner, J.; Kresse, G. Ab Initio Molecular Dynamics for Liquid Metals. *Phys. Rev. B* **1993**, *47*, 558–561.

(32) Hafner, J.; Kresse, G. Ab Initio Molecular-Dynamics Simulation of the Liquid-Metal–Amorphous-Semiconductor Transition in Germanium. *Phys. Rev. B* **1994**, *49*, 14251–14269.

(33) Kresse, G.; J, F. Efficiency of Ab-Initio Total Energy Calculations for Metals and Semiconductors Using a Plane-Wave Basis Set. *Comput. Mater. Sci.* **1996**, *6*, 15–50.

(34) Kresse, G.; J, F. Efficient Iterative Schemes for Ab Initio Total-Energy Calculations Using a Plane-Wave Basis Set. *Phys. Rev. B* **1996**, *54*, 11169–11186.

(35) Hammer, B.; Hansen, L.; Nørskov, J. Improved adsorption energetics within density-functional theory using revised Perdew-Burke-Ernzerhof functionals. *Phys. Rev. B* **1999**, *59*, 7413–7421.

(36) Blochl, P. E. Projector augmented-wave method. *Phys. Rev. B* **1994**, *50*, 17953–17979.

(37) Monkhorst, H. J.; Pack, J. D. Special points for Brillonin-zone integrations. *Phys. Rev. B* **1976**, *13*, 5188–5192.

(38) Larsen, A. H. et al. The Atomic Simulation Environment-A Python library for working with atoms. *J. Phys. Condens. Matter* **2017**, *29*, 273002.

(39) Brogaard, R. Y.; Henry, R.; Schuurman, Y.; Medford, A. J.; Moses, P. G.; Beato, P.; Svelle, S.; Nørskov, J. K.; Olsbye, U. Methanol-to-hydrocarbons conversion: The alkene methylation pathway. *J. Catal.* **2014**, *314*, 159–169.

(40) Rossmeisl, J.; Qu, Z. W.; Zhu, H.; Kroes, G. J.; Nørskov, J. K. Electrolysis of water on oxide surfaces. *J. Electroanal. Chemi.* **2007**, *607*, 83–89.

(41) Henkelman, G.; Uberuaga, B. P.; Jónsson, H. A climbing image nudged elastic band method for finding saddle points and minimum energy paths. *J. Chem. Phys.* **2000**, *113*, 9901–9904.

(42) Giannozzi, P. et al. QUANTUM ESPRESSO: A modular and open-source software project for quantum simulations of materials. *J. Phys.: Condens. Matter* **2009**, *21*, 395502–395521.

(43) Katsounaros, I.; Cherevko, S.; Zeradjanin, A. R.; Mayrhofer, K. J. J. Oxygen electrochemistry as a cornerstone for sustainable energy conversion. *Angew. Chem. Int. Ed.* **2014**, *53*, 102–121.

(44) Burke, M. S.; Enman, L. J.; Batchellor, A. S.; Zou, S.; Boettcher, S. W. Oxygen Evolution Reaction Electrocatalysis on Transition Metal Oxides and (Oxy)hydroxides: Activity Trends and Design Principles. *Chem. Mater.* **2015**, *27*, 7549–7558.

(45) Tahir, M.; Pan, L.; Idrees, F.; Zhang, X.; Wang, L.; Zou, J. J.; Wang, Z. L. Electrocatalytic oxygen evolution reaction for energy conversion and storage: A comprehensive review. *Nano Ener.* **2017**, *37*, 136–157.

(46) Song, J.; Wei, C.; Huang, Z. F.; Liu, C.; Zeng, L.; Wang, X.; Xu, Z. J. A review on fundamentals for designing oxygen evolution electrocatalysts. *Chem. Soc. Rev.* **2020**, *49*, 2196–2214.

(47) Lee, Y.; Suntivich, J.; May, K. J.; Perry, E. E.; Shao-Horn, Y. Synthesis and activities of rutile IrO2 and RuO2 nanoparticles for oxygen evolution in acid and alkaline solutions. *J. Phys. Chem.Lett* **2012**, *3*, 399–404.

(48) Stoerzinger, K. A.; Qiao, L.; Biegalski, M. D.; Shao-Horn, Y. Orientation-dependent oxygen evolution activities of rutile IrO2 and RuO2. *J. Phys. Chem. Lett.* **2014**, *5*, 1636–1641.

(49) Nong, H. N.; Oh, H.-s.; Reier, T.; Willinger, E.; Willinger, M.-g.; Petkov, V.; Teschner, D.; Strasser, P. Oxide-supported IrNiOx core–shell particles as efficient, cost-effective, and stable catalysts for electrochemical water splitting. *Angew. Chem. Int. Ed.* **2015**, *54*, 2975–2979.

(50) Bernicke, M.; Ortel, E.; Bergmann, A.; Ferreira De Araujo, J.; Strasser, P.; Kraehnert, R. Iridium oxide coatings with templated porosity as highly active oxygen evolution catalysts: Structure-activity relationships. *ChemSusChem* **2015**, *8*, 1908–1915.

(51) García-Melchor, M.; Vilella, L.; López, N.; Vojvodic, A. Computationally probing the performance of hybrid, heterogeneous, and homogeneous Iridium-based catalysts for water oxidation. *ChemCatChem* **2016**, *8*, 1792–1798.

(52) Man, I. C.; Su, H. Y.; Calle-Vallejo, F.; Hansen, H. A.; Martínez, J. I.; Inoglu, N. G.; Kitchin, J.; Jaramillo, T. F.; Nørskov, J. K.; Rossmeisl, J. Universality in Oxygen Evolution Electrocatalysis on Oxide Surfaces. *ChemCatChem* **2011**, *3*, 1159–1165.

(53) Seh, Z. W.; Kibsgaard, J.; Dickens, C. F.; Chorkendorff, I.; Nørskov, J. K.; Jaramillo, T. F. Combining theory and experiment in electrocatalysis: Insights into materials design. *Science* **2017**, *355*, eaad4998.

(54) Gunasooriya, G. T. K.; Nørskov, J. K. Analysis of Acid-Stable and Active Oxides for the Oxygen Evolution Reaction. *ACS Ener. Lett.* **2020**, *5*, 3778–3787.

(55) Viswanathan, V.; Hansen, H. A.; Nørskov, J. K. Selective electrochemical generation of hydrogen peroxide from water oxidation. *J. Phys. Chem. Lett.* **2016**, *6*, 4224–4228.

(56) Siahrostami, S.; Li, G. L.; Viswanathan, V.; Nørskov, J. K. One- or Two-Electron Water Oxidation, Hydroxyl Radical, or H2O2 Evolution. *J. Phys. Chem. Lett.* **2017**, *8*, 1157–1160.

(57) Siahrostami, S.; Vojvodic, A. Influence of adsorbed water on the oxygen evolution reaction on oxides. *J. Phys. Chem. C* **2015**, *119*, 1032–1037.

(58) Liu, L. M.; Zhang, C.; Thornton, G.; Michaelides, A. Structure and dynamics of liquid water on rutile TiO2(110). *Phys. Rev. B Cond. Mat. Mater. Phys.* **2010**, *82*, 161415–161419.
